# Simulation of calcium signaling in fine astrocytic processes: Effect of spatial properties on spontaneous activity

**DOI:** 10.1371/journal.pcbi.1006795

**Published:** 2019-08-19

**Authors:** Audrey Denizot, Misa Arizono, U. Valentin Nägerl, Hédi Soula, Hugues Berry

**Affiliations:** 1 INRIA, F-69603, Villeurbanne, France; 2 Univ Lyon, LIRIS, UMR5205 CNRS, F-69621, Villeurbanne, France; 3 Interdisciplinary Institute for Neuroscience, Université de Bordeaux, Bordeaux, France; 4 Interdisciplinary Institute for Neuroscience, CNRS UMR 5297, Bordeaux, France; 5 Univ P&M Curie, CRC, INSERM UMRS 1138, F-75006, Paris, France; University of Geneva, SWITZERLAND

## Abstract

Astrocytes, a glial cell type of the central nervous system, have emerged as detectors and regulators of neuronal information processing. Astrocyte excitability resides in transient variations of free cytosolic calcium concentration over a range of temporal and spatial scales, from sub-microdomains to waves propagating throughout the cell. Despite extensive experimental approaches, it is not clear how these signals are transmitted to and integrated within an astrocyte. The localization of the main molecular actors and the geometry of the system, including the spatial organization of calcium channels *IP*_3_*R*, are deemed essential. However, as most calcium signals occur in astrocytic ramifications that are too fine to be resolved by conventional light microscopy, most of those spatial data are unknown and computational modeling remains the only methodology to study this issue. Here, we propose an *IP*_3_*R*-mediated calcium signaling model for dynamics in such small sub-cellular volumes. To account for the expected stochasticity and low copy numbers, our model is both spatially explicit and particle-based. Extensive simulations show that spontaneous calcium signals arise in the model via the interplay between excitability and stochasticity. The model reproduces the main forms of calcium signals and indicates that their frequency crucially depends on the spatial organization of the *IP*_3_*R* channels. Importantly, we show that two processes expressing exactly the same calcium channels can display different types of calcium signals depending on the spatial organization of the channels. Our model with realistic process volume and calcium concentrations successfully reproduces spontaneous calcium signals that we measured in calcium micro-domains with confocal microscopy and predicts that local variations of calcium indicators might contribute to the diversity of calcium signals observed in astrocytes. To our knowledge, this model is the first model suited to investigate calcium dynamics in fine astrocytic processes and to propose plausible mechanisms responsible for their variability.

## Author summary

Astrocytes process information in the brain via calcium signals that can modulate neuronal communication. Astrocytic calcium signals are associated with brain functioning, including memory and learning, and are altered in the diseased brain. Astrocytic calcium signals display a huge spatio-temporal diversity, which mechanisms and functional roles are poorly understood. 80% of calcium signals occur in the gliapil, corresponding to astrocytic ramifications that are too thin to be detected by conventional light microscopy. Because of the small volumes at stake, we modeled astrocytic calcium signals in the gliapil with a stochastic spatially-explicit individual-based model. Our model successfully reproduces calcium signals that we measured in hippocampal astrocytic gliapil and sheds light to the importance of the localization of calcium sources. We predict that the diversity of calcium signals measured with fluorescent indicators might be partly due to local variations of the concentration of those indicators. We believe that this model will be useful to investigate the propagation of calcium signals within the sponge-like network of astrocytic processes, and eventually to better understand information processing in the brain.

## Introduction

Astrocytes were first characterized as non-excitable cells of the central nervous system since, although they express voltage-gated channels [1], they do not exhibit electrical excitability [2]. Astrocytes excitability instead results from variations of cytosolic calcium concentration [3]. At the cellular level, those calcium signals emerge in astrocytes in response to synaptic activity and may cause the release of molecules called gliotransmitters such as glutamate, ATP, tumor necrosis factor-*α*, or D-serine, which can modulate synaptic transmission [4–7] and vasoconstriction/vasodilatation [8–11]. This close association of astrocytes to pre- and post- synaptic elements, both structurally and functionally, is referred to as tripartite synapse (see e.g. [12–15] for reviews on tripartite synapses and the associated controversies). On a larger scale, astrocytic calcium signals can modulate neuronal synchronization and firing pattern [16–18] and have been observed *in vivo* in response to external stimuli [19, 20]. Altogether, those observations disrupt the traditional view that allocates information processing in the brain to neurons only.

Cell culture, *ex vivo* and *in vivo* studies have demonstrated that astrocytes display both spontaneous calcium signals [19, 21–25] and neuronal activity-induced calcium signals [17, 20, 26]. Astrocytic calcium signals can be localized to synapses [4, 26–28], propagate along processes [29], lead to whole-cell events [30] or even propagate to other cells [31]. Whether this spatio-temporal variability of calcium signals is associated to different physiological functions and whether this could reflect signal integration from different neural circuits is still unknown.

Astrocytic calcium signals are considered to rely mainly on the *IP*_3_*R* calcium channel pathway. Indeed, type-2 *IP*_3_*R* calcium channel is enriched in astrocytes [32] and knocking-out *IP*_3_*R*2 channels abolishes all calcium signals in astrocytic soma and roughly half of them in the cell processes [28]. The molecular origin of the *IP*_3_*R*2-independent signals in processes remains a matter of debate, and could involve calcium fluxes through the plasma membrane [28] and/or other *IP*_3_*R* channel subtypes [33]. In any case, astrocytes respond to G-protein-coupled receptor (GPCR) agonists with calcium transients [34, 35]. Binding of agonists to G_q/11_-GPCRs activates *IP*_3_ synthesis. In turn, binding of both *IP*_3_ and calcium ions to *IP*_3_*R* channels on the membrane of the endoplasmic reticulum (ER) triggers a calcium influx from the ER to the cytosol [36]. The initiation and propagation of calcium signals within astrocytes then relies on the so-called calcium-induced-calcium release (CICR) mechanism: an increase, even small, of the local calcium concentration increases *IP*_3_*R* opening probability thus increasing the probability for local calcium concentration to rise further.

80% of the astrocyte calcium activity *in vivo* take place in the gliapil, which is mostly formed by astrocytic ramifications that cannot be spatially resolved by conventional light microscopy [37], yet account for 75% of the astrocytic volume [38]. According to electron microscopy studies, the perisynaptic astrocyte projections (PAPs) that belong to the gliapil could be as thin as 30-50nm in diameter [39, 40]. At this spatial scale, calcium signals are characterized by non-uniform spatial distributions composed of hotspots where calcium signals are more likely to occur and repeat [41, 42]. Those observations suggest the existence of subcellular spatial organizations responsible for the spatial distribution of calcium signal patterns. Understanding calcium signaling in PAPs, where astrocytes potentially regulate neuronal information processing, is crucial. However, only calcium signals in thicker processes, around 300nm in diameter, are within reach of current conventional imaging methods [40] and most studies on astrocytic calcium have focused on astrocytic soma and main processes, where characteristics and physiological roles of calcium signals are likely to differ from those of PAPs. Because of the small dimensions and volumes at stake, modeling is currently the only approach that can investigate calcium signal generation, transmission and the effect of spatial properties within PAPs.

Mathematical models of CICR-based signaling date back to the beginning of the 1990s (for recent reviews see e.g. [43–45]). The first *IP*_3_*R*-mediated calcium signaling models assumed perfect mixing of the molecular species and deterministic kinetics (ordinary differential equations) and typically treated *IP*_3_ concentration as a parameter [46–48]. In those models, calcium transients emerge as limit-cycle oscillations from a Hopf bifurcation (or a saddle-node on an invariant circle) beyond a critical value of the *IP*_3_ concentration. The first astrocyte-specific calcium signaling models arose a decade later. In those models, the *IP*_3_ concentration is usually a dynamical variable coupled to calcium but calcium transients still emerge through the Hopf-bifurcation scenario. Notably, those models focused on intercellular *IP*_3_ transport within astrocyte networks via gap junctions [49, 50]. Stochastic models of *IP*_3_*R*-mediated calcium signaling have also been proposed, that take into account the stochasticity associated with molecular interactions [51–54]. Yet, none of those studies accounts both for molecular species diffusion and stochasticity of the reactions taking place inside astrocytes, which is essential for modeling the stochastic effects associated with small volumes and the low copy number of molecules or ions involved in fine processes. Recently, individual-based modeling has been introduced to evaluate the impact of diffusive noise on *IP*_3_*R* opening dynamics [55], but this simplified model disregarded *IP*_3_ dynamics and restricted stochasticity to the vicinity of the *IP*_3_*Rs*.

Here, we propose an *IP*_3_*R*-mediated calcium signaling model adapted to the dynamics of CICR in small spatial volumes corresponding to thin PAPs. To account for the stochasticity inherent to small sub-cellular volumes and low copy numbers expected in fine processes, our model is both spatially explicit and particle-based: each molecule is described individually, diffuses in space through a random walk and reacts stochastically upon collision with reaction partners. The kinetics of *IP*_3_*R* channels is accounted for with a simplified version of the 8-state Markov model on which most of the previous CICR models are based. In order to explore the range of dynamical behaviors that the model can display, we first focus on a 2D version of our model, that is less compute-intensive than the 3D version. Extensive simulations of the 2D model show that spontaneous calcium signals arise in the model via the interplay between the excitability of the system and its stochasticity. The model accounts for various forms of calcium signals (“blips” and “puffs”) and their frequency depends on the spatial organization of the *IP*_3_*R* channels. In particular, we demonstrate that the co-localization of sources of calcium influx plays a crucial role in triggering an effect of *IP*_3_*R* clustering on calcium signaling. Finally, as solute concentrations can hardly be defined in 2D, we use a 3D version of the model in order to compare it to experimental data. We show that the spontaneous calcium signals generated by the 3D model with realistic process volume and astrocytic calcium concentrations successfully reproduce the spontaneous calcium transients measured in calcium micro-domains with confocal microscopy in organotypic culture of hippocampal astrocytes. Our simulations predict that local variations of the concentration of calcium indicators such as GECIs might contribute to the diversity of calcium signals observed in astrocytes so that precise monitoring of their concentration should be performed. Our model therefore represents the first validated tool to investigate calcium signals in realistic small sub-cellular volumes such as in PAPs, where astrocytes and synapses communicate. This provides a crucial step towards a better understanding of the spatiotemporal response patterns of astrocytes to neuronal activity and beyond, towards astrocyte-neuron communication.

## Results

### Spontaneous oscillations in the model

**Fig 1:**
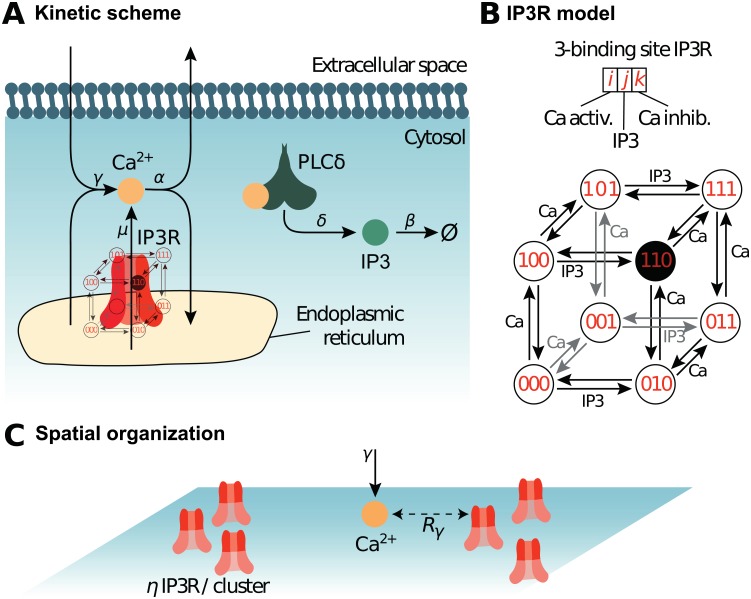
Reaction scheme and *IP*_3_*R* model. The biochemical processes included in the model are illustrated in (*A*). Cytosolic calcium can exit the cytosol to the extracellular space or the endoplasmic reticulum (ER) at a (total) rate *α*, lumping together the effects of ER and plasma membrane pumps. Likewise, Ca^2+^ can enter the cytosol from the extracellular space or from the ER via *IP*_3_*R*-independent flow, with (total) rate *γ*, emulating calcium channels from the plasma membrane. When an *IP*_3_*R* channel opens, calcium enters the cytosol through the channel at rate *μ*. Phospholipase C*δ* (PLC*δ*), once activated by calcium binding, produces *IP*_3_ at rate *δ*. Like Ca^2+^, *IP*_3_ can bind the *IP*_3_*R* channel and is removed with rate *β*. (*B*) Our model of the kinetics of the *IP*_3_*R* channel is an 8-state Markov model adapted from [46, 56]. Each *IP*_3_*R* channel monomer is associated with 3 binding sites, two calcium binding sites and one *IP*_3_ binding site. Occupancy states are designated by a triplet {*i*, *j*, *k*} where *i* stands for the occupation of the first Ca binding site (*i* = 1 if bound, 0 else), *j* for that of the *IP*_3_ binding site and *k* for the second Ca site. The first calcium binding site has higher affinity than the second. The open state is state {110}, where the first Ca and the *IP*_3_ sites are bound but not the second Ca site. (*C*) Spatial parameters for the particle-based model. The $N_{{\text{IP}}_{\text{3}}\text{R}}\;IP_{\text{3}}R$ molecules are positioned within uniformly distributed clusters, with *η* *IP*_3_*R* in each cluster. Hence *η* = 1 corresponds to uniformly distributed *IP*_3_*R* (no clustering), while the degree of clustering increases with *η* (for constant total *IP*_3_*R* number). To account for potential co-localization between *IP*_3_*R*-dependent and *IP*_3_*R*-independent calcium sources, the influx of *IP*_3_*R*-independent calcium (at rate *γ*) occurs within distance *R*_*γ*_ of an *IP*_3_*R*. Thus, low values of *R*_*γ*_ emulate co-localization between *IP*_3_*R*-dependent and *IP*_3_*R*-independent Ca^2+^ influx sources.

We first analyzed our particle-based model for the CICR signaling system of Fig 1, with parameter values presented in Table 1. To that end, we compared Monte-Carlo simulations of the particle-based model in two dimensions with the corresponding Mean-Field and Gillespie’s SSA models (see Methods section). Those three models represent different levels of approximation: the Mean-Field model assumes deterministic kinetics and perfect mixing; the SSA model keeps the perfect mixing hypothesis but assumes stochastic kinetics while the particle-based model assumes stochastic kinetics but accounts for potential non-perfect mixing, i.e. diffusion effects. For comparison with SSA, we first considered perfect mixing of Ca^2+^ ions and *IP*_3_ molecules in the particle-based model by setting the diffusion coefficients *D*_Ca_ = *D*_IP3_ = ∞ (see Method section).

**Table 1:** Parameter values and initial conditions of the 2D model. a.u: arbitrary unit. In 2d, by definition, a MC time unit is 100 Δ*t* and one MC space unit is set by the interaction radius of *IP*_3_*R*, i.e. $d_{{\text{IP}}_{\text{3}}\text{R}}=1.0\;\text{MC}$ space unit. *δ*, *β*, *μ*, *γ*, *b*_1_, *b*_2_ and *b*_3_ are first order constants, in (MC time unit)^−1^. Diffusion coefficients *D*_Ca_ and *D*_IP3_ are expressed in (MC space unit)^2^.(MC time unit)^−1^ whereas *α*, a_1_, a_2_, a_3_ are expressed in (MC space unit)^2^.(MC time unit)^−1^.

Parameter	Description	Value in 2d model
V	Cell volume	200 × 200 a.u.
*IP*_3_ dynamics		
*IP*_0_	Initial *IP*_3_ number/conc.	15 molec.
*D*_IP3_	*IP*_3_ diffusion	10 a.u
*N*_plc_	PLC*δ* number/conc.	1000 molec.
*δ*	PLC*δ* max rate	0.1 a.u
*β*	*IP*_3_ decay	0.01 a.u
Ca^2+^ dynamics		
*Ca*_0_	Initial Ca^2+^ number/conc.	50 molec.
*D*_Ca_	Ca^2+^ diffusion	varied
*μ*	Ca^2+^ flux through open *IP*_3_*R*	50 a.u
*γ*	cytosolic Ca^2+^ influx	50 a.u
*α*	Ca^2+^ decay rate	1.0 a.u
*IP*_3_*R*		
$N_{{\text{IP}}_{\text{3}}\text{R}}$	*IP*_3_*R* number	1000 molec.
$d_{{\text{IP}}_{\text{3}}\text{R}}$	*IP*_3_*R* interact. distance	1 space unit
*IP*_3_*R* binding		
*a*_1_	First Ca	1.0 a.u
*a*_2_	*IP*_3_	1.0 a.u
*a*_3_	Second Ca	0.1 a.u
*IP*_3_*R* dissociation		
*b*_1_	First Ca	0.1 a.u
*b*_2_	*IP*_3_	0.1 a.u
*b*_3_	Second Ca	0.1 a.u

**Fig 2:**
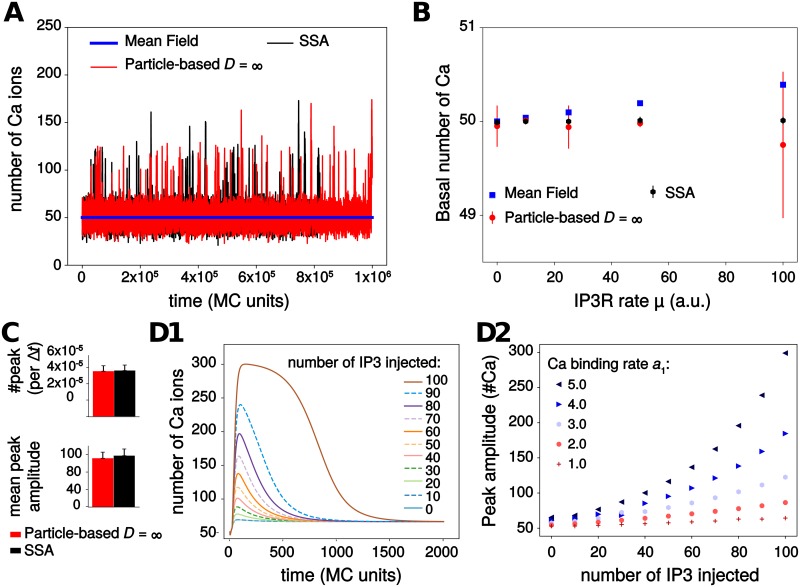
Model exploration. (*A*) Spontaneous transients are observed in simulations of the particle-based and the Gillespie’s SSA model but not in the Mean Field model. (*B*) The three models display the same basal calcium level when *μ*, the calcium influx rate through open *IP*_3_*R* channels, increases. The higher variability in the stochastic models reflects the integer value of basal calcium (either 49 or 50, depending on simulations). (*C*) Quantification of calcium transients in the stochastic models (calcium peak frequency and mean peak amplitude). No significant difference between the two models was observed. (*D*) Excitability of the Mean-Field model: increasing quantities of exogenous *IP*_3_ molecules were injected at time *t* = 20Δ*t*, after model equilibration. The amplitude of the resulting calcium response (*D1*) was quantified depending on the amount of *IP*_3_ injected and the value of the binding rate constant to the first calcium *IP*_3_*R* site, *a*_1_ (*D2*). Parameter values for the particle-based model: *D*_Ca_ = *D*_IP3_ = ∞ (perfect mixing) and *η* = 1, *R*_*γ*_ = 200, i.e. no *IP*_3_*R* channels clustering, and no co-localization of *IP*_3_*R* with *IP*_3_*R*-independent Ca^2+^ sources. For SSA and particle-based models, the figure shows the average ± standard deviation over 20 simulations.

Fig 2A shows one simulation sample for each model. A first result is that the stochastic models (SSA and particle-based) do exhibit spontaneous calcium peaks with the parameters of this figure. On top of a background level of approximately 50 Ca^2+^ ions, with fluctuations of roughly ± 20 ions, large and fast peaks arise spontaneously with a total amplitude between 20 and 120 ions above the baseline. In strong opposition, the (deterministic) mean-field model does not show these oscillations: one gets a stationary trace, that systematically coincides with the baseline level of the stochastic traces (Fig 2B). Comparing the two stochastic models (SSA and particle-based) indicates that both display the same basal calcium level (Fig 2B) and the same frequency and mean peak amplitude (Fig 2C). Altogether, this suggests that stochasticity is necessary for spontaneous calcium signals to occur in this model.

We next searched for the dynamical mechanism that gives rise to those spontaneous peaks. A thorough numerical parameter exploration of the mean-field model failed to demonstrate the existence of Hopf bifurcations or of any other bifurcation that would generate limit-cycle oscillations in the model. This is a distinctive feature of our model, since spontaneous oscillations in the vast majority of *IP*_3_*R*-mediated calcium signaling models arise from limit-cycle generating bifurcations [46–48]. This is however not unexpected since the simplifications made to derive our model significantly reduced its nonlinearity compared to these models, and the emergence of limit-cycle bifurcations demands strong nonlinearity. For instance, limit-cycle oscillations in the classical Li and Rinzel model [48] disappear when *IP*_3_*R* opening needs less than three open monomers. However, our model retains enough nonlinearity to exhibit excitability. To demonstrate this, we used the mean-field model, waited until all concentrations reached their stationary state, and injected an increasing amount of exogenous *IP*_3_ molecules. In response to this *IP*_3_ injection, a calcium transient was obtained, before relaxation to the stationary state (Fig 2D). Fig 2D2 shows how the resulting transient amplitude depends on the amount of injected *IP*_3_. For low values of *IP*_3_*R* calcium binding rate (first site), *a*_1_, the calcium response is basically linear with the number of injected *IP*_3_: doubling the amount of *IP*_3_ injected only doubles the amplitude of the calcium response. However, as *a*_1_ increases, peak amplitude becomes a strongly nonlinear function of the number of *IP*_3_ injected. With *a*_1_ = 5 a.u. for instance, doubling the number of injected *IP*_3_ from 50 to 100 results in an almost threefold increase of the calcium response. Therefore the mean-field model with large values of *a*_1_ is an excitable system that amplifies the fluctuations of *IP*_3_ in its calcium responses. We conclude that spontaneous calcium transients occur in the system of Fig 1 through the interplay of the stochasticity of the SSA or particle-based models and the underlying excitability of the system.

### Transitions between calcium activity regimes

The experimental and modeling literature on intracellular calcium signals distinguishes two classes of localized calcium peaks: puffs and blips [57]. Blips refer to brief and weak peaks that correspond to the opening of a single *IP*_3_*R* channel (or a single *IP*_3_*R* channel tetramer), whereas puffs are longer and higher peaks resulting from the concerted opening of a group of nearby *IP*_3_*R* channels (or tetramers thereof), via the calcium-induced calcium-release principle. We next examined whether our model was able to reproduce these observations.

**Fig 3:**
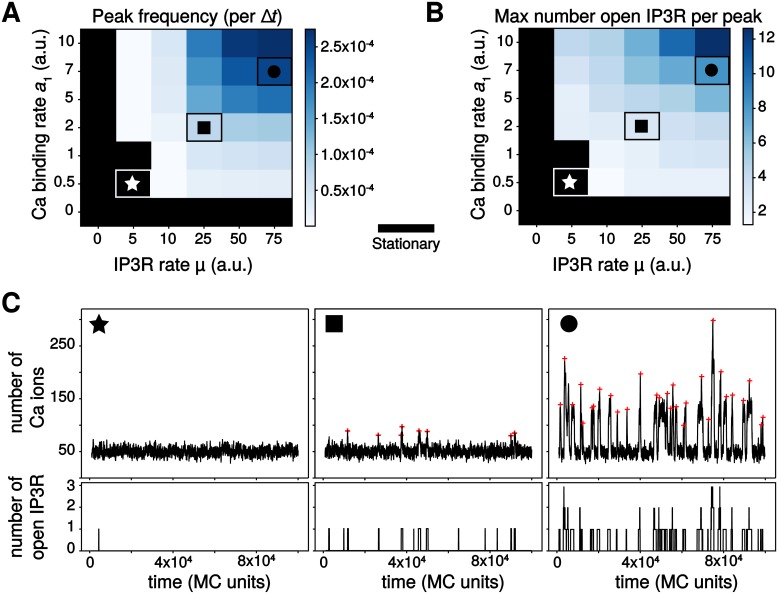
The particle-based model produces different calcium activity regimes depending on parameter values. Color-coded map of variation of the peak frequency, expressed as the number of calcium peaks per MC time step (*A*) and as the maximal number of *IP*_3_*R* channel open per peak (*B*). The color scale is given for each map. The black area corresponds to the stationary regime. Note that the *x* and *y*-axis scales in (*A*) and (*B*) are not regularly spaced. The symbols ★, ◼ and ● locate parameter pairs that are illustrative of the three dynamical regimes shown in (*C*): stationary (★, *μ* = 5, *a*_1_ = 0.5), blips (◼, *μ* = 25, *a*_1_ = 2) and puffs (●, *μ* = 75, *a*_1_ = 7). Red crosses show the locations of peaks from automatic detection. *D*_Ca_ = *D*_IP3_ = ∞, *η* = 1, *R*_*γ*_ = 200.

We carried out parameter exploration of the particle-based model in conditions of perfect mixing for mobile molecules (*Ca* and *IP*_3_) and uniform spatial distribution of the immobile ones (PLC*δ*, *IP*_3_*R*). As expected, we found that calcium peaks frequency depends on parameter values (Fig 3A). When the rate of calcium influx through open *IP*_3_*R* channels *μ* or the binding rate constant to the first Ca *IP*_3_*R* site *a*_1_ are too small, the model does not exhibit calcium peaks at all, only fluctuations around a stationary state (Fig 3C★). This is in agreement with our analysis of the system excitability above, that evidenced excitability only for large enough values of *a*_1_ (Fig 2D2). Note however that in the model, *IP*_3_*R* openings do not necessarily lead to a calcium peak, especially for low values of both *μ* and *a*_1_ (Fig 3C★). Spontaneous calcium transients are obtained in the particle-based model beyond threshold of (*μ*, *a*_1_) values, with a peak frequency that increases with parameters values (Fig 3A). Inspection of the maximal number of open *IP*_3_*R* per peak reveals that not only the frequency, but also the type of these transient signals changes with parameters values (Fig 3A): the less frequent signals are generally associated with a single open *IP*_3_*R* per peak (Fig 3C◼), corresponding to blips, whereas the high-frequency spontaneous signals rely on the opening of 2 − 12 *IP*_3_*R* in a peak (Fig 3C●), corresponding to puffs. In agreement with experimental observations [58, 59], calcium puffs in the particle-based model are characterized by higher peak amplitude and peak duration compared to blips. Taken together, these results show that our particle-based model not only reproduces the existence of spontaneous calcium peaks in conditions of low copy numbers, it is also able to reproduce the existence of different types of localized calcium transients, in agreement with experimental measurements.

### Impact of calcium diffusion coefficient on calcium signals

A modeling study has demonstrated the necessity to account for the stochasticity inherent to calcium diffusion when modeling calcium signaling in small volumes [60]. We next investigated the impact of calcium diffusion on calcium dynamics in the particle-based model. In neurons or astrocytes, the amount of endogenous calcium buffers is large so that the diffusion distance of free calcium is believed to be very small. Many of the endogenous buffers are however mobile. Buffers can have a very significant effect on calcium dynamics because they decrease the diffusion distance and the effective diffusion coefficient of calcium ions [53, 54, 61–64]. Here, we have chosen not to include buffers explicitly in the model for the sake of model simplicity, but to account for their presence by decreasing the diffusion coefficient for calcium. Therefore, the latter is to be interpreted as an effective diffusion coefficient lumping together calcium buffering by mobile endogenous buffer and diffusion of these buffers. To confirm that explicit addition of buffers yields effects similar to a decrease of the *Ca*^2+^ diffusion coefficient, we have explicitly added endogenous buffer molecules to our 2D model in a subset of simulations, assigning a low coefficient of diffusion for buffers and high one for free calcium ions. These simulations confirmed the absence of significant difference between simulations obtained using fast calcium diffusion and slow explicit buffers on the one hand, and our reference model without buffers but with an effective lower *D*_Ca_ on the other hand (S1 Fig).

Moreover, several plasma membrane proteins, in particular the Na^+^-Ca^2+^ exchanger (NCX) have been observed to co-localize with ER proteins in neurons and astrocytes [65]. Such a co-localization of calcium signaling molecules might imply spatial organizations including raft-like micro-domains. This organization seems essential for calcium wave propagation in astrocytes [66]. Moreover, mGluR5-ER proteins co-clusters mediated by an interaction with Homer1 scaffold protein have been observed in astrocytic processes [67]. Homer1 is also known for increasing calcium activity in neurons by increasing *IP*_3_*R*-mGluR5 proximity [68]. Those experimental studies suggest that several calcium sources are co-localized with ER proteins in astrocytes and that it might alter calcium dynamics. Such a co-localization could be crucial for calcium signaling, in particular in small volumes. We thus placed our study of the influence of calcium mobility on calcium signaling in a framework where calcium sources (*IP*_3_*R*-dependent and *IP*_3_*R*-independent) can co-localize.

To this end, the *IP*_3_*R*-independent calcium influx in the cytosol (from e.g. plasma membrane transporters or channels) was made dependent on parameter *R*_*γ*_, that sets the distance from *IP*_3_*R* receptors within which new calcium ions are injected in the cytosol when they originate from *IP*_3_*R*-independent fluxes (see Methods section). When *R*_*γ*_ = 0, the initial location of the new calcium ion is shared with an *IP*_3_*R* channel whereas when *R*_*γ*_ increases, the injection positions of new calcium ions are increasingly uncorrelated from those of the *IP*_3_*R* channels. When *R*_*γ*_ becomes as large as the size of the reaction surface (i.e. for *R*_*γ*_ = 100), the injection position of the new calcium ion is effectively independent of the positions of the *IP*_3_*R* channels.

**Fig 4:**
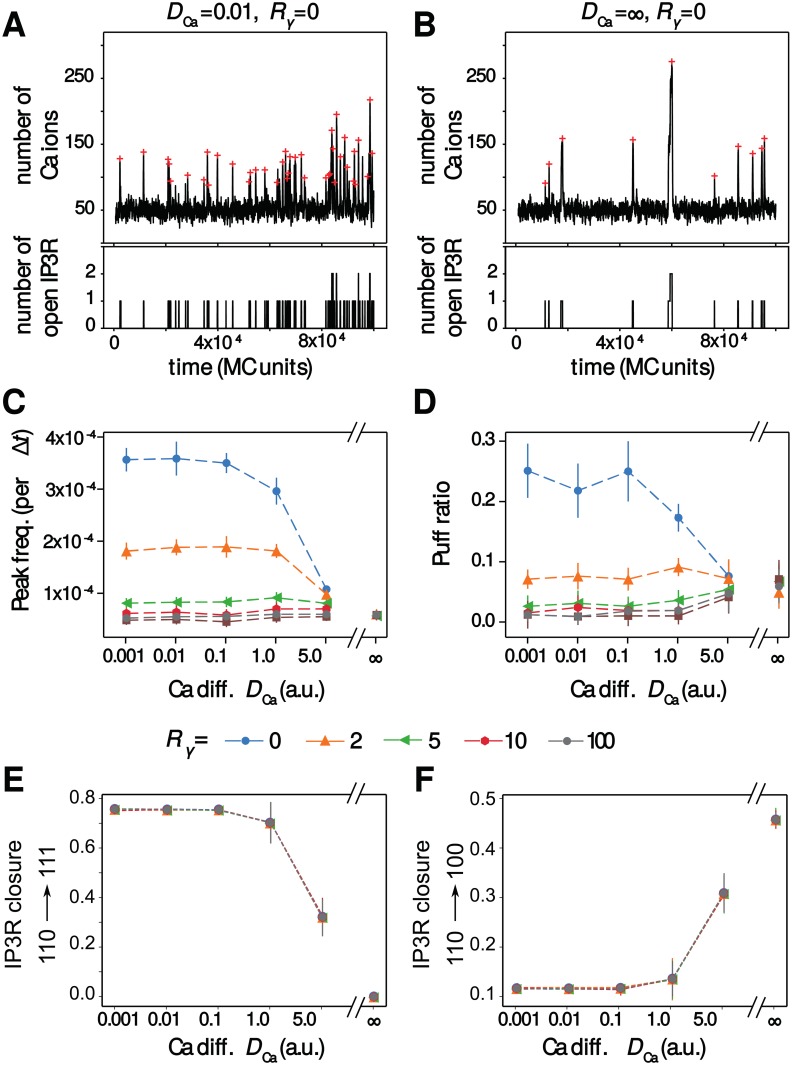
Ca^2+^ diffusion modulates the temporal characteristics of the signals upon co-localization. Representative simulations of the particle-based model showing both calcium trace and number of open *IP*_3_*R* for co-localized calcium sources (*R*_*γ*_ = 0) in the case of slow calcium diffusion (*A*) or perfect-mixing of calcium (*B*). The red crosses show peak locations from automatic detection. The impact of calcium diffusion coefficient *D*_Ca_ on peak frequency (*C*) and the amount of puff (*D*) are shown for different values of the co-localization parameter *R*_*γ*_: from *R*_*γ*_ = 0 (*IP*_3_*R* are not clustered but co-localized with other calcium sources) to *R*_*γ*_ = 100 (*IP*_3_*R* are neither clustered nor co-localized). The puff ratio quantifies the fraction of peaks that are puffs. (*E*) and (*F*) respectively present the probabilities that *IP*_3_*R* closure results from binding of a *Ca*^2+^ to the inactivating site (probability to switch to state {111}, *P*_110−>111_) or unbinding of an *IP*_3_ (probability to switch to state {100}, *P*_110−>100_) depending on *D*_Ca_ and on *R*_*γ*_. Probability of closure due to *Ca*^2+^ unbinding from activating site, *P*_110−>010_ can be deduced from 1 = *P*_110−>010_ + *P*_110−>100_+*P*_110−>111_. Data are presented as mean ± standard deviation over 20 simulations. Lines are guide for the eyes. Note that the *x*-axis scale in (*C*), (*D*), (*E*) and (*F*) is not regularly spaced. Other parameters: *η* = 1 (no clustering), *a*_1_ = 1 a.u, *μ* = 50 a.u.

Our simulations show that the impact of the calcium diffusion coefficient is mainly visible when calcium sources are co-localized, i.e. for small values of *R*_*γ*_. Fig 4A and 4B compare a representative simulation obtained when Ca^2+^ diffuses slowly (A) with a simulation obtained with perfectly-mixed calcium (B), in a case where the *IP*_3_*R* receptors are not clustered (*η* = 1). Those representative simulations hint that the peak frequency is much larger with slow calcium, and suggests that slow calcium diffusion slightly favors the puff regime compared to perfect mixing. The systematic quantification of Fig 4C and 4D confirms these interpretations: when *IP*_3_*R*-dependent and *IP*_3_*R*-independent calcium sources are co-localized, i.e. for *R*_*γ*_ < 5, the value of *D*_Ca_ controls calcium transient frequency, as well as the probability to observe a puff. The effects are strong: for instance for *R*_*γ*_ = 0, decreasing *D*_Ca_ from 5 to 0.1 increases the frequency roughly threefold. However, when the *IP*_3_*R*-independent influx was not co-localized with *IP*_3_*R* channels (i.e. for *R*_*γ*_ ≥ 5), both the peak frequency and the type of signal were found not to depend on the calcium diffusion coefficient anymore. Those results suggest that calcium diffusion could control the frequency and type of calcium signals within astrocytes when *IP*_3_*R* channels are co-localized with *IP*_3_*R*-independent calcium sources.

Once open, i.e in state {110}, the *IP*_3_*R* can switch to state {111} with probability *P*_110−>111_, due to binding of *Ca*^2+^ to the inactivating site. Open receptors can also switch to state {100} (or {010}) with probability *P*_110−>100_ (or *P*_110−>010_, respectively), due to the unbinding of *IP*_3_ (or of *Ca*^2+^, respectively) from the activating site. Fig 4E and 4F shows how the probabilities *P*_110−>111_ and *P*_110−>100_ vary with *D*_Ca_ and *R*_*γ*_ (*P*_110−>010_ can be deduced from 1 = *P*_110−>010_ + *P*_110−>100_+*P*_110−>111_). In contrast, *R*_*γ*_ has no significant effect on *P*_110−>111_, *P*_110−>100_ and *P*_110−>010_ probabilities. The effect of the effective diffusion coefficient *D*_Ca_ is strong: when low, most of *IP*_3_*R* closure is due to the binding of *Ca*^2+^ to the inhibiting site. As *D*_Ca_ increases, *P*_110−>111_ decreases and in well-mixed conditions (*D*_Ca_ = ∞), *IP*_3_*R* closure is always due to the stochastic unbinding of *IP*_3_ and *Ca*^2+^. So, receptor closure is strongly dominated by binding of *Ca*^2+^ to the inactivating site when *Ca*^2+^ effective diffusion is slow, but mostly relies on unbinding from the activating sites for fast *Ca*^2+^ effective diffusion. This result illustrates that well-mixed simulations are not well-suited to study the self-inhibiting behaviour of *IP*_3_*R*, i.e the fact that the *Ca*^2+^ influx resulting from the opening of a given *IP*_3_*R* can subsequently shut down this very receptor. Therefore accounting for diffusion with spatial models appears necessary to the study of the dynamics of *IP*_3_*R* at the single-receptor scale.

### *IP*_3_*R* clustering controls calcium signals when co-localized

**Fig 5:**
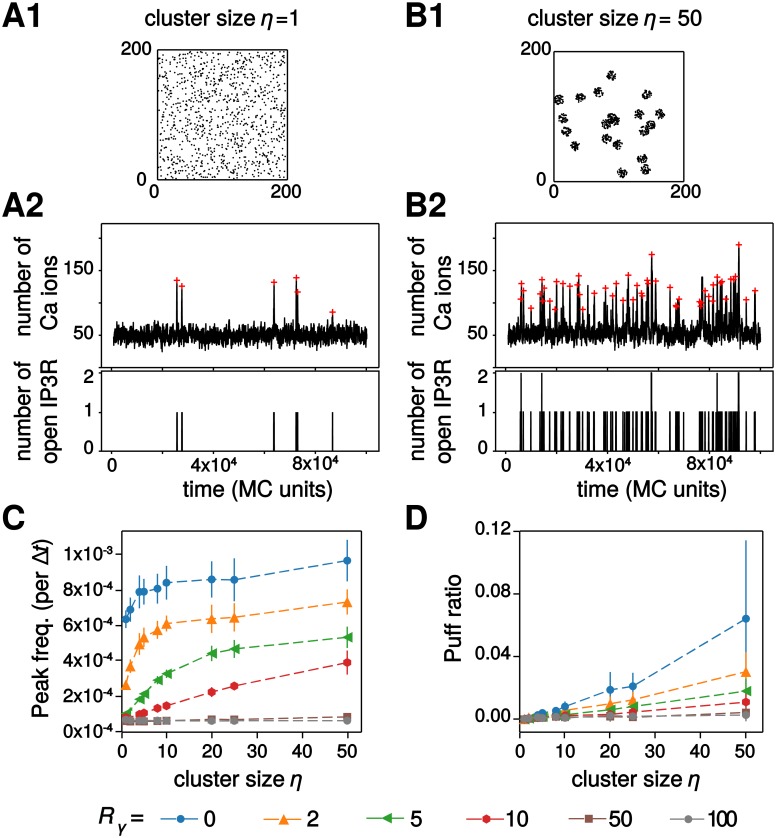
*IP*_3_*R* clustering modulates calcium signals when co-localized. Representative simulations of the particle-based model with the corresponding *IP*_3_*R* distribution over space, the calcium trace and number of open *IP*_3_*R* for weakly co-localized calcium sources (*R*_*γ*_ = 10) in the case of uniform distribution of the *IP*_3_*R* (*A*) or strongly clustered *IP*_3_*R* (*B*) are illustrated. The red crosses show peak locations from automatic detection. The impact of *IP*_3_*R* cluster size *η* on calcium peak frequency (*C*) and on the amount of puffs (*D*) are shown for different values of the cluster size: from *η* = 1 (*IP*_3_*R* are not clustered) to *η* = 50 (strong clustering). Data are presented as mean ± standard deviation over 20 simulations. Lines are guide for the eyes. Other parameters: *D*_Ca_ = 0.1 a.u, *a*_1_ = 1 a.u, *μ* = 50 a.u.

Experimental data demonstrate that *IP*_3_*R* in SH-SY5Y and COS7 cells are not uniformly distributed on the ER membrane but form clusters [58, 59]. We next investigated the impact of *IP*_3_*R* clustering on calcium signal dynamics in our particle-based model. Simulations were performed with *D*_Ca_ = 0.1 and various amounts of co-localization between *IP*_3_*R* channels and other calcium sources (parameter *R*_*γ*_). Representative simulations for uniformly-distributed *IP*_3_*R* channels (*η* = 1) and strongly clustered *IP*_3_*R* (*η* = 50) are presented in Fig 5A and 5B. In these two examples, the *IP*_3_*R* were weakly co-localized with the *IP*_3_-independent calcium sources (i.e. *R*_*γ*_ = 10). These traces indicate that the frequency and type of calcium signal in this case is heavily dependent on the spatial distribution of *IP*_3_*R* channels: clustered *IP*_3_*R* seem to exhibit much larger peak frequency and slightly more frequent puffs. However, here again this effect is quite mitigated by the amount of co-localization between *IP*_3_*R* channels and the *IP*_3_*R*-independent calcium sources. In particular, the dynamical range of the modulation by *IP*_3_*R* cluster size *η* (i.e. the ratio between the frequency at *η* = 50 and *η* = 1) is maximal for intermediate co-localizations (2 ≤ *R*_*γ*_ ≤ 10) but the calcium peak frequency is hardly dependent on *η* when co-localization is either very strong (*R*_*γ*_ < 2) or very weak (*R*_*γ*_ ≥ 50). Increasing clustering also tends to improve the emergence of puffs, although the effect is significant only for strong co-localization (*R*_*γ*_ ≤ 2, Fig 5D). We emphasize that in such cases of strong co-localization, the regime of calcium activity (puffs *vs* blips) changes by simply rearranging the spatial distribution of the *IP*_3_*R*, without changing any of the kinetics parameters of the model.

Taken together, these simulation results pinpoint the interplay between calcium source co-localization and the degree of *IP*_3_*R* clustering as a crucial modulator of temporal characteristics of the calcium signals and of the signaling regime. In particular, they suggest that in the presence of certain amount of co-localization between *IP*_3_*R* channels and other sources of calcium influx in the cytosol the spontaneous calcium peak frequency can have a large amplitude variation. Within this range of parameters, calcium peak frequency can be finely tuned by the geometry of the colocalization.

### Simulations in a compartmentalized 3d geometry reproduce spontaneous calcium microdomains signals

**Fig 6:**
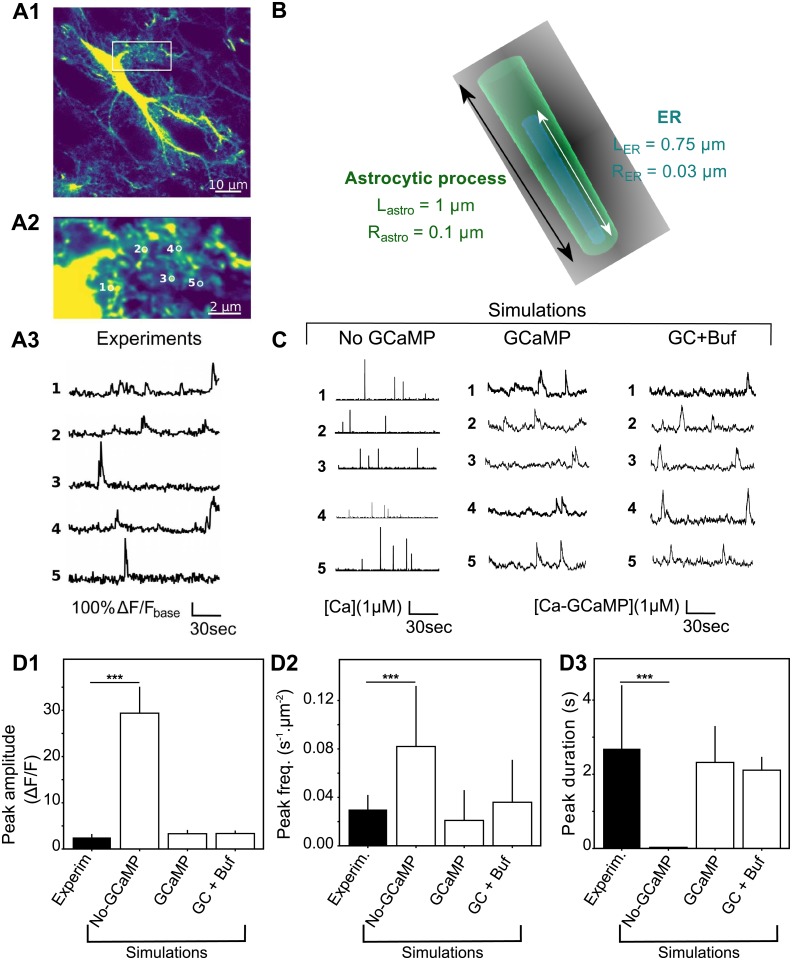
3d model simulations in fine astrocyte processes successfully reproduce calcium microdomains signals. (*A*) Experimental monitoring of the spontaneous local Ca^2+^ signals in astrocytic sponge-like processes. Panel *A1* shows a ‘summed projection’ of a confocal time lapse image stack of a GCaMP6s-expressing astrocyte. Panel *A2* illustrates magnification of the boxed region of panel *A1*. Panel *A3* displays spontaneous calcium traces from the regions of interest shown in (*A2*). (*B*) The 3d geometry used for the 3D model is a cylinder of length *L*_astro_ = 1 *μ*m and radius *R*_astro_ = 0.1 *μ*m, with ER as a thinner cylinder inside. The interior volume is roughly 0.03 fL. (*C*) Representative simulations of calcium dynamics within the above cylinder with the “No-GCaMP”, “GCaMP” and “GC+Buf” simulations. The raw signal corresponds to cytosolic free calcium concentration for the “No-GCaMP” model and to calcium-bound GCaMP concentration for “GCaMP” and “GC+Buf” models. For all simulation types, parameter values were partly taken from the literature and partly adjusted for fitting calcium traces shown in *A* (reported in Table 2). (*D*) Quantitative comparisons of the spontaneous calcium signals measured experimentally (black bars) or simulated with the “No-GCaMP”, “GCaMP” or “GC+Buf” models (white bars). The compared quantities are peaks amplitude in terms of Δ*F*/*F* ratio (D1), their frequency (measured in *min*^−1^ for each *μm*^2^ area, D2) and duration (expressed as full width at half maximum, FWHM) D3. Significance is assigned by * for *p* ≤ 0.05, ** for *p* ≤ 0.01, *** for *p* ≤ 0.001.

The above 2d simulations of the particle-based model have the advantage of a good computational efficiency, which makes them suitable for parametric studies with averaging over a number of Monte-Carlo simulations. However, the 2d setting does not facilitate the comparison of the copy number of molecules in the simulations with species concentrations as measured experimentally. Moreover, it is difficult to investigate with a 2d setting the impact of the fact that *IP*_3_*R* channels are specifically localized at the surface of the ER membrane and not freely diffusing in the cytosol bulk. To tackle those questions, we carried out simulations of our model in a more refined three-dimensional setting (Fig 6), in which we could adjust more precisely molecule concentrations, reaction volume and cytosol compartmentalization to what is expected in fine astrocytic processes. We then compared our simulations to experimental measurements of calcium dynamics in microdomains of comparable dimensions in mice hippocampal organotypic culture (Fig 6A). We have chosen to use organotypic slices as they provide better optical access and sample stability, which, combined with confocal microscopy, enabled us to distinguish individual processes (resolution ≈ 200 nm VS ≈ 500 nm with two-photon microscopy *in vivo*). While this resolution is not enough to resolve the exact sizes of PAPs, it provides the most realistic calcium dynamics experimentally available for calcium transients occurring at fine astrocytic processes.

As 80% of calcium activity occurs in astrocytic ramifications that cannot be resolved by optical microscopy [38], astrocytic calcium signaling models must take into account small volumes associated to it. For that purpose, we created the 3d structure mimicking one process geometry shown in Fig 6B. The reaction volume was chosen to match the range of sizes that are within reach of current imaging methods: a 1 *μm*-long cylinder of 100 nm radius (i.e. a volume around 0.03 fL), inside which we position a 0.75 *μm*-long cylindrical ER with a radius of 30 nm. In this 3d implementation, *Ca*^2+^ and *IP*_3_ molecules diffuse in the bulk 3D space located between the external (plasma) membrane and that of the ER, while *IP*_3_*R* molecules are distributed uniformly at random over ER membrane surface.

Our calcium imaging of calcium dynamics in fine astrocyte processes reveals the sponge-like structure of the processes Fig 6A1, with localized submicron calcium microdomains (regions of interest (ROI) in Fig 6A2) of size that can be less than 0.5*μm*^2^. The corresponding calcium traces display infrequent (a few hundredths of Hz) peaks with average amplitude around 2 (Δ*F*/*F*) and typical duration of ≈ 2.7 seconds at FWHM (Fig 6A3 and 6D). Notice that these experimental traces correspond to spontaneous signals to the extent that they were measured in the absence of any neuronal or astrocytic stimulation. In particular, TTX application in this preparation did not alter peak frequency [69].

Our first noticeable result is that our model is able to reproduce the emergence of spontaneous calcium peaks of comparable frequency, duration and signal-to-noise ratio (Fig 6C). This result therefore indicates that spontaneous calcium signals can emerge in the fine processes even with a realistic basal calcium concentration of 83 ± 29 nM, which corresponds to only one to two calcium ions in the whole cylinder. Quantification of the free *Ca*^2+^ signal properties shows that signals are quantitatively and qualitatively different from experimental signals (Fig 6C and 6D, “No-GCaMP” simulations). Adding GCaMP6s to the model improved drastically both qualitatively and quantitatively the match between simulations and experimental data (Fig 6C and 6D, “GCaMP” and “GC+Buf” simulations), with no apparent difference between the “GCaMP” and the “GC+Buf” models. Note that our experimental statistics are tightly associated with the temporal sampling frequency used in the experiments (2 Hz) since very fast calcium events may be accessible only to higher sampling frequencies [38]. In particular, the experimental peak frequency measured might have been higher with better temporal resolution. Our spontaneous signals measured in organotypic hippocampal cultures are of the same order of magnitude as the ones measured *in vivo* [38, 70]. In any case, our results show that genetically encoded calcium indicators (GECIs), such as GCaMP6s, may change local calcium concentration, in particular close to open *IP*_3_*R* channels, leading to an increased peak duration. Those results are in accordance with previous studies that demonstrate that calcium buffers, such as GECIs, modulate signal readout [53, 71].

Together these results demonstrate that our model, without any endogenous buffers, is enough to reproduce calcium signals within fine astrocytic processes in a quantitative way, making it a powerful tool to investigate calcium dynamics in the small volumes associated with PAPs.

### Effect of GCaMP properties on calcium dynamics

**Fig 7:**
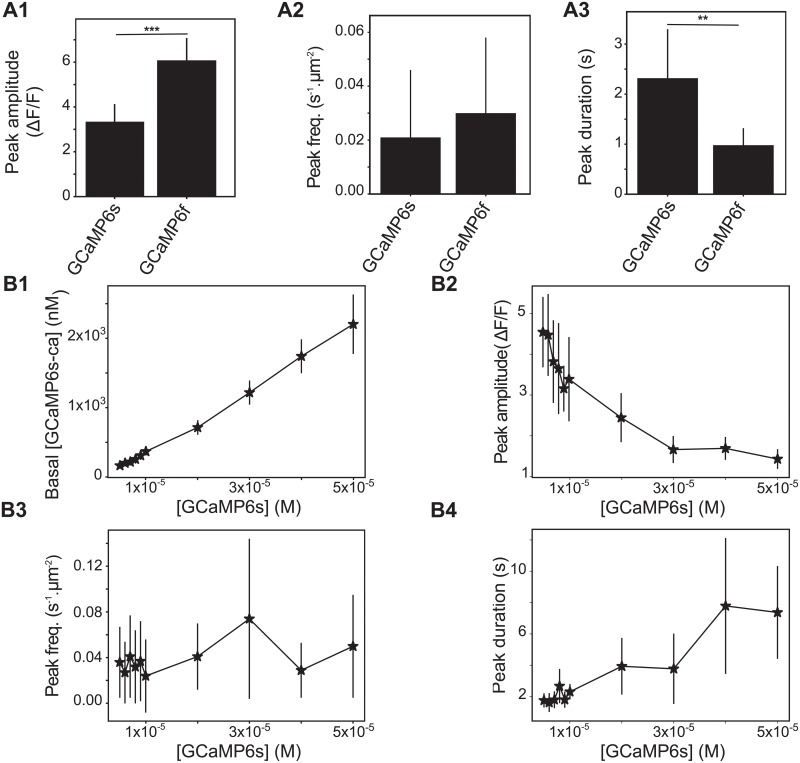
The kinetics and concentration of GECIs strongly influence calcium dynamics. (*A*) Quantitative comparisons of the spontaneous calcium signals measured with “GCaMP6s” or “GCaMP6f” as fluorescent reporters. The compared quantities are peak amplitude in terms of Δ*F*/*F* ratio (A1), frequency (measured in *min*^−1^ for each *μm*^2^ area, A2) and duration (expressed as full width at half maximum, FWHM) (A3). (*B*) The impact of the concentration of GCaMP6s in the system on basal concentration of GCaMP-Ca (B1), on the GCaMP-Ca peak amplitude (B2), frequency (B3) and duration (B4) are shown for different values of [GCaMP6s]. Significance is assigned by * for *p* ≤ 0.05, ** for *p* ≤ 0.01, *** for *p* ≤ 0.001. Data are presented as mean ± standard deviation over 20 simulations. Lines are guide for the eyes.

Because our “GCaMP” simulations revealed that the use of GECIs may change local calcium concentration and thus impact peak duration, we have next investigated the effect on calcium dynamics of several parameters defining GCaMP molecules: their kinetics and their concentration. We tested to what extent using different GECIs in our simulations impacted calcium dynamics. We compared the dynamics of [GCaMP6s-Ca] with those of [GCaMP6f-Ca]. Although the total concentration of GECIs in those two models is identical, GCaMP6f-Ca signals display higher amplitude and smaller duration than GCaMP6s-Ca signals (Fig 7A1 and 7A3). Those results are partially in agreement with experimental measurements [72] that have reported a similar decrease of peak duration when using GCaMP6f compared to GCaMP6s. However, experimental observations also included a decrease of the peak amplitude with GCaMP6f, that we do not observe. This discrepancy could be due to a higher fluorescence baseline of GCaMP6f-Ca in those experiments, leading to decreased Δ*F*/*F* ratio.

As the concentration of GECIs cannot be controlled experimentally and is often not reported in calcium imaging studies, we have next investigated its effect on calcium signals (Fig 7B). Our simulations demonstrate that an increased GCaMP concentration in the cell results in a linear increase of basal GCaMP-Ca levels (Fig 7B1), with an unchanged basal concentration of free calcium. Increased [GCaMP] is associated with a decrease of GCaMP-Ca peak amplitude expressed in terms of Δ*F*/*F* ratio (Fig 7B2) and an increase of peak duration (Fig 7B4). Interestingly, varying [GCaMP] does not seem to have an impact on peak frequency (Fig 7B3), which is contradictory to Skupin et al’s results that have demonstrated a non-linear increase of the average signal period with the concentration of exogenous buffers [54]. However, Skupin et al studied whole-cell EGTA or BAPTA dynamics, which is fundamentally different from the local spontaneous GCaMP-Ca signals in the fine processes that we are modelling here. Local variations of cellular GCaMP concentration might thus yield variations of peak duration and amplitude, so that measuring cellular GCaMP concentration and its variations along the cellular compartments appears crucial to analyze calcium signals more accurately.

## Discussion

Recent experimental reports suggested that the complete dependence of cytosolic calcium transients on *IP*_3_*R*2 is only observed in the astrocyte cell body whereas calcium signals measured within astrocytic processes are a mix of *IP*_3_*R*2-dependent and non-*IP*_3_*R*2-dependent calcium signals [21, 28]. The identity, subtype and localization of the receptors responsible for non-*IP*_3_*R*2-dependent calcium signals in astrocytes, in particular their processes, are still to be uncovered. However, our study sheds light on the importance of the localization of these various calcium sources. Our simulation results indeed indicate that when *IP*_3_*R* channels are (even moderately) co-localized with *IP*_3_*R*-independent calcium sources, e.g. plasma membrane calcium channels, the degree of *IP*_3_*R* clustering and/or the mobility of the calcium buffers will have a strong impact on the frequency and amplitude of the spontaneous calcium signals. In particular, our simulations predict that two astrocyte processes expressing exactly the same repertoire of channels, pumps and receptors but in a different spatial organization (for instance various degrees of clustering or co-localization), can exhibit very different types and properties of spontaneous calcium events. This could result in significant variability of the calcium response of different processes, even from the same cell. Moreover, our results suggest that ‘puffs’ might reflect cellular sub-compartments in which calcium channels are co-localized, which increases the calcium response to a given stimulus. It would thus be interesting to investigate whether those co-localizations can be observed at specific locations, such as at neuron-astrocyte contact sites, or if they are randomly distributed within the cell.

During the past few years, fine astrocytic processes have been regarded as devoid of ER [73, 74]. This questions the validity of our model, in which the presence of ER-attached *IP*_3_*R* in the process is crucial for spontaneous activity. We however note that a recent EM study has observed that ER dynamically ramified in astrocyte perivascular processes *in vivo* and detected contact sites between ER processes and plasma membrane, often positioned in apposition to neuronal synapses [75]. Such contiguous membranous juxtapositions would definitely validate the presence of ER in PAPs. Although dynamical ER remodeling has been reported in dissociated astrocyte culture [76], technical limitations have prevented direct investigation of ER localization within PAPs *in vivo* or in slices. To our knowledge, it is not even clear whether astrocytic ER is continuous or consists in several independent reservoirs. Super-resolution microscopy of cellular ER and mitochondrial dynamics and structure (resolution ≈ 100nm) has recently been developed and could help solve the controversy regarding the presence of ER in fine processes [77, 78]. Correlative super-resolution fluorescence imaging and electron microscopy approaches can yield a resolution of less than 50 nm (down to 10nm) [79], which is very promising avenue to PAPs ultrastructure investigation. ER-bound GECIs, OER-GCaMP6f, have been recently developed and, combined with the use of ER luminal calcium indicators such as G-CEPIA1_*er*_ [80], could help investigate the involvement of calcium channels on the ER membrane in calcium dynamics depending on subcellular localization in astrocytes [81]. In any case, since the *IP*_3_*R* pathway is involved in calcium dynamics, further investigations regarding ER sub-cellular localization, sub-compartmentalization and dynamics are crucial for better understanding astrocyte information processing. Meanwhile, a straightforward extension of our computational model would be to simulate neuronal stimulation-triggered calcium dynamics.

*IP*_3_*Rs* are thought to assemble as tetramers, and a recent experimental study suggested that the four subunits of the tetramer must be simultaneously bound to *IP*_3_ for the tetramer to allow calcium influx, independently of cytosolic calcium or ATP concentrations [82]. Actually, the original *IP*_3_*R* model predicted that subunit cooperativity for calcium binding is also necessary to fit experimental data of *IP*_3_*R* dynamics [46, 48]. Even though the *IP*_3_*R* binding sites for calcium have been characterized, their roles in *IP*_3_*R* dynamics are still poorly understood [83]. The requirement for inter-subunit cooperativity, in which the 4 *IP*_3_ binding sites should simultaneously be bound for the tetramer to open, is expected to hinder the emergence of spontaneous calcium events. In a subset of simulations, we have replaced our non-cooperative *IP*_3_*R* model, in which the binding of a single *IP*_3_ site is enough to open the monomer channel, with the cooperative model proposed by Bicknell and collaborators [84]. With 100 nM basal *IP*_3_ and Ca^2+^ [85, 86], we could not produce spontaneous calcium signals in these conditions, even after a search of the parameter space to locate parameters allowing spontaneous activity with this cooperative model. This issue might reflect a general problem of the De Young Keizer model in discrete particle-based models with low copy number of particles. The De Young Keizer model is based on steady-state experimental data representing averages over time and over channel populations, which proved sufficient to reproduce experimental statistics such as the average open time or the steady-state open probability. However, this model might not be suited to describe behaviors at the level of individual channels and low copy number of particles. More recent models have been proposed that successfully reproduce the evolution with time of the open/close dynamics of a single *IP*_3_R [87, 88]. In those models, the transition rates between different states of the *IP*_3_R are not triggered by Ca^2+^ or *IP*_3_ binding events but by complex continuous functions of their concentrations. We could not implement such complicated functions with a pure particle-based modeling strategy such as used here. Therefore, further investigations are needed to clarify the suitability of the De Young Keizer model in the context of particle-based spatially-explicit stochastic models. Alternatively, our results may be interpreted as casting doubts on the existence of spontaneous calcium signals in astrocytes when the basal *IP*_3_ and Ca^2+^ concentrations are of the order of 100 nM. A number of studies have reported higher calcium concentration localized at the vicinity of calcium channels [89–91]. Such calcium microdomains, in the vicinity of *IP*_3_*R*, could facilitate the emergence of spontaneous signals from cooperative *IP*_3_*Rs* in thin processes.

On the other hand, experimental evidence for spontaneous calcium signals in astrocytes is still debated. Even in the absence of presynaptic neural activity, presynaptic axon terminals do probabilistically release neurotransmitter vesicles, generating so-called miniature EPSCs. Bafilomycin application has been used in several experimental studies to investigate the dependence of astrocytic calcium signals on EPSCs, because this inhibitor of V-ATPases inhibits miniature EPSCs by blocking the refill of presynaptic vesicles. However, the impact of bafilomycin bath application on the frequency of spontaneous calcium signals in astrocytes has proven variable (compare e.g. [92] and [93]). In our preparation, bath-application of bafilomycin strongly decreased peak frequency and amplitude [69]. As bafilomycin has a wide range of effects on calcium signaling that is independent of its effect on the refill of presynaptic neurotransmitter vesicles [94, 95], we cannot conclude whether those signals are triggered by EPSCs and further investigation is needed to decipher whether the “spontaneous” calcium signals reported in astrocyte processes are due to spontaneous release of presynaptic vesicles or rely on a synapse-independent mechanism inherent to the CICR system.

For simplicity, *IP*_3_*R* clustering in our model was considered static during simulation time. Experimentally, though, *IP*_3_*R* clustering might be highly dynamic [96, 97]. Several molecules can trigger *IP*_3_*R* clustering, including *IP*_3_ and calcium themselves [96, 97], through a mechanism that may include the lateral diffusion of *IP*_3_*R* on the ER surface [97] or be independent from it [98]. Beyond this *IP*_3_*R* classification into clustered and un-clustered populations, another approach is to quantify single *IP*_3_*R* channels based on their mobility. A recent study on HeLa cells [41] indicates that calcium signals emerge most of the time from immobile *IP*_3_*R*, which are found in apposition to ER-plasma membrane junctions, whereas the mobile *IP*_3_*R* fraction would not be involved in calcium influx. Our simulation results, in agreement with previous *IP*_3_*R*-mediated calcium models [99, 100], indicate that *IP*_3_*R* clustering can lead to an increase of the frequency and amplitude of their calcium signals. This result is in contradiction with a previous modeling study that concluded in favor of a reduction of *IP*_3_*R* channel activity upon *IP*_3_*R* clustering [101]. This discrepancy might rely on the different modeling choices. In particular, the model in [101] incorporates a 5-state *IP*_3_*R* model derived from Tu et al. [102, 103]. All of those modeling studies however agree that dynamical *IP*_3_*R* clustering could be a mechanism used by astrocyte processes to modulate their calcium signals. This could provide astrocyte processes with a capacity for information processing plasticity.

In our model, the value of the rate constant for calcium binding on *IP*_3_*R* changes the type of spontaneous dynamics (e.g. blips vs puffs) in addition to its characteristics (frequency, amplitude). Experimentally, several post-transcriptional mechanisms can modulate *IP*_3_*R* affinity. For instance, phosphorylation of type-1 and -2 *IP*_3_*R* by cAMP-activated PKA increases the affinity of *IP*_3_*R* to calcium and *IP*_3_ [104]. At a larger time scale, the sensitivity of *IP*_3_*R* to calcium is encoded in a sequence of calcium sensor (Cas) region that differs depending on the *IP*_3_*R* isoform [102, 105, 106]. Since multiple *IP*_3_*R* isoforms seem to be involved in calcium signaling within astrocytic processes [33], they could assemble into a variety of homo- or hetero- *IP*_3_*R* tetramers that would exhibit a range of calcium and *IP*_3_ affinity.

In addition, immobile or weakly mobile endogenous calcium buffers are responsible for an effective intracellular calcium diffusion that is an order of magnitude slower than free calcium ions [107]. Our simulation results indicate that the value of the effective Ca^2+^ mobility also participates in the determination of the type and characteristics of the spontaneous events, thus confirming previous experimental [108] and modeling studies [53, 60, 62, 109, 110]. Although our simulations with both GCaMP and endogenous buffers,”GC+Buf”, overall displayed dynamics similar to the simulations without endogenous buffers (”GCaMP”), we note that, similarly to the effect of GCaMP concentration, increasing the concentration of endogenous buffers led to longer duration of the calcium signals. Those results are consistent with previous studies that have demonstrated significant effects of buffers [61] or of intra-cluster channel communication and coupling [53] on calcium dynamics. Endogenous calcium buffers display various kinetics and diffusion coefficients in astrocytes [111] and some of them are overexpressed in hippocampal and striatal astrocytes, possibly in a region-specific pattern [112], which could be involved in the regional variability of astrocytic calcium signals [113]. Our study shows that precisely accounting for the effects of GECIs and endogenous calcium buffers on calcium dynamics is crucial for better interpreting calcium signals in PAPs. Particular care should be taken when interpreting GCaMP-Ca signals as GCaMP concentration is rarely monitored although it could be partly responsible for the diversity of calcium signals observed in PAPs.

Therefore, in addition to the spatial organization of the Ca^2+^ channels, the differential expression of endogenous calcium buffers, including the fluorescent Ca^2+^ reporters, could also be potential determinants allowing a range of responsiveness and spatio-temporal characteristics of calcium signals in astrocyte processes.

To conclude, we have presented a spatially-explicit stochastic model to investigate intracellular calcium signaling based on CICR in small sub-cellular volumes. Recent studies proposed models for the simulation of astrocytic sodium [114] and calcium signals [74, 115, 116] in 3d with deterministic differential equation models that correspond to cellular volumes large enough to validate a law of large numbers. To our knowledge, our model is the first model suited to reproduce spontaneous calcium signals in the finest astrocyte processes, where low copy number and spatial localization effects are expected to be more prominent than in larger volumes. Our simulations demonstrate that low copy number of molecules can display dynamics that cannot be predicted by deterministic approaches and that spatial modelling is crucial to better understand the effect of molecular distributions and sub-compartments geometries on calcium dynamics. Since these fine processes are thought to be the place of initiation of neuron-astrocyte interactions, we believe that this model, combined with models of signal propagation between astrocytic compartments such as Savtchenko et al. [116], might be useful to investigate the initiation and spatiotemporal integration of calcium signals in the sponge-like network of astrocyte processes, a prerequisite to understand neuron-astrocyte communication.

## Materials and methods

### Modeling methods

#### Reaction scheme

The model considers cytosolic calcium and *IP*_3_ dynamics in the framework of calcium-induced calcium release (CICR) signaling. The reaction scheme considered is shown in Fig 1 A. In short, we consider calcium fluxes between the cytosol and the extracellular space or the endoplasmic reticulum (ER), including via *IP*_3_*R* channels. We also take into account the effect of phospholipase C *δ* (PLC*δ*), that, when activated by calcium, synthesizes *IP*_3_. To derive simple models for this scheme, we made the following assumptions:

- We considered that the extracellular and ER calcium concentrations are constant during the simulation, as well as the electrical potentials across the plasma and ER membranes. In this case, calcium outflow from the cytosol to the ER or to the extracellular medium can be lumped into a single first-order rate *α*. Likewise, calcium entry from the extracellular medium or any *IP*_3_*R*-independent Ca^2+^ influx from the ER can be considered constants, too. We lumped them into a single overall constant flux *γ*.
- PLC*δ* enzymes remain located in the cytosol (no translocation) and the amount of their substrate PIP2 is present everywhere in large excess. Under this condition, activated PLC*δ* produces *IP*_3_ with constant rate *δ*.

*IP*_3_*R* channels are gated both by calcium and *IP*_3_, with a bell-shaped dependence of the open probability to calcium concentration [56]. To model their dynamics, we used the classical 8-state Markov model proposed in [46, 56], with two calcium binding sites, referred to as ‘Ca sites’, and one *IP*_3_ binding site for each *IP*_3_*R* (see Fig 1B). However we used the following simplifications:

- We considered that the binding or unbinding rate constant of a given binding site is independent from the occupancy state of the other sites (no intra-channel cooperativity). Under this assumption, the rate constant for calcium binding at the first calcium binding site, *a*_1_, does not depend on whether the other two binding sites are bound or not. Thus, the rate constant for {000} + *Ca* → {100} has the same value as e.g. the reaction {011} + *Ca* → {111} (where the triplet notation corresponds to the one defined in Fig 1). Likewise, the rate constants for Ca^2+^ or *IP*_3_ binding or unbinding to the three sites were considered independent from the other occupancy states.
- The open state is assumed to be state {110} (first Ca site and *IP*_3_ bound, second Ca site free), as in [46, 56]. These latter models further assume inter-channel cooperativity, where *IP*_3_*R* channels assemble as tetramers of which at least three monomers must be in the open state for calcium to be transferred. Here we neglected inter-channel cooperativity and considered that every single channel was open when in the open state i.e., as long as an *IP*_3_*R* channel is open, it is assumed to inject calcium in the cytosol at constant rate *μ*.

#### Monte Carlo simulations of the spatially-explicit stochastic particle-based model

We first modeled the kinetic scheme described in Fig 1 with a lattice-free spatially-explicit stochastic particle-based model, referred to as “Particle-based” model below, in two spatial dimensions, with reflective boundary conditions. Each molecule of the system was explicitly modeled with its associated position in space. PLC*δ* and *IP*_3_*R* molecules were considered immobile whereas Ca^2+^ and *IP*_3_ molecules were mobile by diffusion. At the beginning of each Monte-Carlo (MC) simulation of this model, the space coordinates for each Ca^2+^, *IP*_3_ and PLC*δ* molecules are chosen uniformly at random.

To determine the positions of the $N_{{\text{IP}}_{\text{3}}\text{R}}\;IP_{\text{3}}R$ molecules, we first chose the centers of $N_{c}=N_{{\text{IP}}_{\text{3}}\text{R}}/\eta \;IP_{\text{3}}R$ clusters uniformly at random in the reaction space, where *η* is the number of *IP*_3_*R* per cluster (as illustrated in Fig 1C). For each cluster, we positioned *η* *IP*_3_*R* molecules uniformly at random within a distance *R*_*c*_ of the cluster center, with $R_{c}=d_{IP_{3}R}\sqrt{\eta /0.91}$, where $d_{{\text{IP}}_{\text{3}}\text{R}}$ is the interaction distance of the *IP*_3_*R*, i.e. the maximal distance between *IP*_3_*R* center and a Ca^2+^ or *IP*_3_ molecule below which binding can occur. According to this algorithm, *η* = 1 corresponds to randomly distributed independent *IP*_3_*R* molecules (no clustering) whereas *IP*_3_*R* molecules become increasingly clustered when *η* increases, with constant *IP*_3_*R* density within the clusters and constant total *IP*_3_*R* number in the reaction space.

Each MC stimulation step (of duration Δ*t*) consists in iterating the following steps:

1. *Diffusion*. The position of each mobile molecule (Ca^2+^ and *IP*_3_) is updated independently according to Brownian motion: $\text{r}(t+\Delta t)=\text{r}(t)+\sqrt{2D_{i}\Delta t}\xi$, where *D*_*i*_, *i* = {Ca, IP3} is molecule *i* diffusion coefficient and *ξ* is a vector of i.i.d. Gaussian-distributed random numbers with zero mean and unit variance. In a subset of simulations, the new position of each mobile molecule was chosen at random in the reaction volume, i.e. **r**(*t* + Δ*t*) = *ζ* were *ζ* is a vector of i.i.d. random numbers uniformly distributed in [0, *L*], with *L* the length of the spatial domain. We refer to this setting as “infinite” diffusion coefficients, *D* = ∞.
2. *Binding*. For each Ca^2+^ ion close enough to a PLC*δ* to react (i.e. when the distance between both is less than the interaction radius of PLC*δ*), a new *IP*_3_ molecule is created with probability *δ*Δ*t* at the position of the PLC*δ* molecule. Likewise, each Ca^2+^ or *IP*_3_ molecule close enough to an *IP*_3_*R* molecule (i.e. within its interaction radius) can bind it depending on its occupancy state. If the *IP*_3_ binding site is free, an *IP*_3_ molecule binds with probability *a*_2_Δ*t*. If one of the Ca sites is free, a Ca^2+^ ion binds the free site with probability *a*_1_Δ*t* (first Ca site) or *a*_3_Δ*t* (second Ca site). If both Ca sites are free, binding occurs to the first site with probability *a*_1_Δ*t* and to the second one with probability (1 − *a*_1_Δ*t*)*a*_3_Δ*t*.
3. *Unbinding*. Each *IP*_3_*R* molecule releases its bound Ca^2+^ or *IP*_3_ molecules independently, with probability *b*_1_Δ*t* (first Ca site), *b*_2_Δ*t* (*IP*_3_ site) and *b*_3_Δ*t* (second Ca site). Ca^2+^ or *IP*_3_ molecules that bound the *IP*_3_*R* at the previous (binding) step of the current time step do not unbind.
4. *Removal*. Free cytosolic Ca^2+^ and *IP*_3_ molecules are removed from the cytosol with probability *α*Δ*t* and *β*Δ*t*, respectively. Ca^2+^ and *IP*_3_ molecules that unbound from *IP*_3_*R* at the previous (unbinding) step of the current time step are not removed.
5. *Ca^2+^ Influx*. For each *IP*_3_*R* channel in the open state {110}, a new Ca^2+^ ion is created in the cytosol at the *IP*_3_*R* position with probability *μ*Δ*t*. A new calcium ion can also be created in the cytosol with probability *γ*Δ*t*, mimicking Ca^2+^ influx from *IP*_3_*R*-independent sources in the ER membrane or through the plasma membrane. Note that the position of this new calcium is not uniform over space but depends on parameter *R*_*γ*_ and works as follows: an *IP*_3_*R* molecule is chosen (uniformly) at random and the new Ca^2+^ ion is positioned uniformly at random within distance *R*_*γ*_ of the chosen *IP*_3_*R*. Therefore low values of *R*_*γ*_ emulate co-localization between *IP*_3_*R*-dependent and *IP*_3_*R*-independent Ca^2+^ influx sources, whereas the location of *IP*_3_*R*-independent Ca^2+^ influx is uniform over the reaction volume when *R*_*γ*_ becomes as large as the volume side length.

Table 1 gives the parameter values used in our 2D simulations, including the initial numbers of Ca^2+^, PLC*δ*, *IP*_3_ and *IP*_3_*R* molecules.

#### Mean-field (MF) dynamics of the perfectly stirred model

With infinite diffusion, the dynamics of the system can be assumed to be perfectly stirred. With that mean-field (MF) assumption, the temporal dynamics of reaction scheme Fig 1 can be modeled using ordinary differential equations based on the mass-action law. *IP*_3_*R* dynamics in these conditions can be described with seven ODEs that express the temporal dynamics of the concentration of *IP*_3_*R* in state {*ijk*}, [*ijk*]:

$$\{\begin{array}{l} \text{d}[000]/\text{d}t=-\left(a_{1}[Ca]+a_{2}[IP3]+a_{3}[Ca]\right)[000]+b_{1}[100]+b_{2}[010]+b_{3}[001]\\ \text{d}[001]/\text{d}t=-\left(a_{1}[Ca]+a_{2}[IP3]+b_{3}\right)[001]+b_{1}[101]+b_{2}[011]+a_{3}[Ca][000]\\ \text{d}[010]/\text{d}t=-\left(a_{1}[Ca]+b_{2}+a_{3}[Ca]\right)[010]+b_{1}[110]+a_{2}[IP3][000]+b_{3}[011]\\ \text{d}[011]/\text{d}t=-\left(a_{1}[Ca]+b_{2}+b_{3}\right)[011]+b_{1}[111]+a_{2}[IP3][001]+a_{3}[Ca][010]\\ \text{d}[100]/\text{d}t=-\left(b_{1}+a_{2}[IP3]+a_{3}[Ca]\right)[100]+a_{1}[Ca][000]+b_{2}[110]+b_{3}[101]\\ \text{d}[101]/\text{d}t=-\left(b_{1}+a_{2}[IP3]+b_{3}\right)[101]+a_{1}[Ca][001]+b_{2}[111]+a_{3}[Ca][100]\\ \text{d}[110]/\text{d}t=-\left(b_{1}+b_{2}+a_{3}[Ca]\right)[110]+a_{1}[Ca][010]+a_{2}[IP3][100]+b_{3}[111] \end{array}$$ (1)

where [*Ca*] and [*IP*3] denote Ca^2+^ and *IP*_3_ concentration, respectively. The concentration of the eighth occupancy state, {111} is obtained from conservation of the *IP*_3_*R*, i.e. $\left[111\right]=N_{{\text{IP}}_{3}\text{R}}/V-\left(\left[000\right]+\left[001\right]+\left[010\right]+\left[011\right]+\left[100\right]+\left[101\right]+\left[110\right]\right)$. *IP*_3_ dynamics in the mean-field model is given by:

$$\begin{array}{c} d\left[IP3\right]/dt=-a_{2}\left[IP3\right]\sum_{i=0}^{1}\sum_{k=0}^{1}\left[i0k\right]+b_{2}\sum_{i=0}^{1}\sum_{k=0}^{1}\left[i1k\right]+\delta \left[PLC\delta \right]\left[Ca\right]-\beta \left[IP3\right] \end{array}$$ (2)

where [*PLCδ*] = *N*_plc_/*V*. Finally, the mean-field dynamics of the free Ca^2+^ is obtained with:

$$\begin{array}{c} \begin{array}{cc} d[Ca]/dt= & -(a_{1}\sum_{j=0}^{1}\sum_{k=0}^{1}\left[0jk\right]+a_{3}\sum_{i=0}^{1}\sum_{j=0}^{1}\left[ij0\right])\left[Ca\right]\\ & +b_{1}\sum_{j=0}^{1}\sum_{k=0}^{1}\left[1jk\right]+b_{3}\sum_{i=0}^{1}\sum_{j=0}^{1}\left[ij1\right]\\ & -\alpha [Ca]+\gamma +\mu [110] \end{array} \end{array}$$ (3)

For comparison with the output of the other models, the concentrations were transformed into numbers of molecules by multiplication by the reaction volume *V*.

#### Perfectly-stirred stochastic temporal dynamics (SSA)

For comparison, we also modeled the reaction scheme depicted in Fig 1 using Gillespie’s exact Stochastic Simulation Algorithm (SSA) that accounts for stochasticity due to low copy numbers and assumes perfect mixing of the reactants [117, 118]. Here, the dynamic variables are the number of Ca^2+^ and *IP*_3_ molecules in the system, *N*_Ca_ and *N*_IP3_ and the number of *IP*_3_*R* channels in state {*ijk*}, *N*_ijk_. The rates of all the reactions of the scheme of Fig 1 are then calculated according to mass-action laws like in the MF model of Eqs (1), (2) and (3). For instance, at reaction time *t*, the rate of reaction {001} + *Ca* → {101} is given by *a*_1_/*V N*_001_(*t*)*N*_Ca_(*t*). The next reaction time *τ* is sampled from an exponential distribution with mean 1/*R*_*T*_, where *R*_*T*_ is the sum of the reaction rates of all reactions. The next reaction to occur at time *t* + *τ* is chosen as an integer random variable with point probability given by the ratio of its rate to *R*_*T*_. For instance, for the reaction illustrated above, the probability that this reaction is the one occurring at time *t* + *τ* is *a*_1_/*V N*_001_(*t*)*N*_Ca_(*t*)/*R*_*T*_. Finally, the variables are updated according to the chosen reaction. In the data presented below, we have modeled each receptor individually, i.e. for each receptor $l\in 0\ldots N_{{\text{IP}}_{3}\text{R}}$, $N_{ijk}^{l}=1$ if receptor *l* is in state *ijk*, 0 else. If the illustration reaction described above on receptor *l* is chosen, this means $N_{001}^{l}(t+\tau )=N_{001}^{l}(t)-1$, *N*_Ca_(*t* + *τ*) = *N*_Ca_(*t*) − 1 and $N_{101}^{l}(t+\tau )=N_{101}^{l}(t)+1$. The other variables keep their values.

#### Realistic simulations in 3d astrocytic processes

In order to simulate calcium dynamics within a more refined 3d geometry with realistic volumes and concentrations, we built a model in STEPS (http://steps.sourceforge.net/). STEPS is a software for voxel-based stochastic reaction-diffusion simulations within complex 3d geometries that simulates stochastic chemical reaction-diffusion with a spatialized version of Gillespie’s SSA, usually referred to as the reaction-diffusion master equation (RDME). In RDME, space is partitioned into voxels inside which perfect mixing is assumed, while diffusion between adjacent voxels is modeled as first order reactions [119, 120]. STEPS uses a derivative of the SSA in tetrahedral voxels that allows for a better resolution than the cubic voxels mostly used in voxel-based models [121].

*Geometry*. The main advantage of STEPS in the context of the present study is its automatic handling of external and internal membranes [122]. Moreover, STEPS simulations can easily be parallelized [123], a crucial property given the computational burden of such compartmentalized 3d simulations. This allowed us to explicitly describe the presence of the ER membrane inside the 3d cell cytoplasm and the fact that *IP*_3_*R* channels are located in the ER membrane. The geometry of the reaction volume consisted in a cylinder of length *L*_astro_ = 1 *μ*m and radius *R*_astro_ = 0.1 *μ*m. The ER was modeled as a second cylinder, internal, with length *L*_ER_ = 0.75 *μ*m and radius *R*_ER_ = 0.03 *μ*m. The resulting cytosolic volume (2.81 × 10^−17^ L) was meshed with 11345 tetrahedra of individual volume 2.48 × 10^−21^ *L*, thus ensuring the well-mixed subvolume condition [121].

*Reactions*. In this spatial configuration, we modeled the *IP*_3_*R*-mediated calcium signaling kinetic scheme of Fig 1 with *IP*_3_*R* channels positioned on the intracellular ER membrane and according to three model variants:

- A first variant, referred to as the “No-GCaMP” model, did not include fluorescent calcium indicators. In this 3d model, parameter values were taken, whenever possible, from the literature (Table 2). *γ* and *α* values were adjusted to yield basal calcium concentration around 120 nM [86, 124]. Likewise, *β* and *μ* were adjusted for a basal *IP*_3_ concentration of 120nM [85]. Note that this value is based on recent, precise measurements of *IP*_3_ concentration and differs by an order of magnitude from *IP*_3_ concentration values routinely used in *IP*_3_*R*-mediated calcium models [59, 125, 126]. *IP*_3_*R* density on the ER surface has been measured from TIRF-microscopy analysis in cell cultures [89], reporting *IP*_3_*R* cluster diameters of 0.3 *μ*m at most, with up to 10 *IP*_3_*R* per cluster. The ER surface area in our model is 0.69 *μ*m^2^. Ignoring the potential unclustered *IP*_3_*Rs* [98], this represents a maximum of 4 clusters, thus at most 40 *IP*_3_*R*. We thus set the number of *IP*_3_*R* in our model to 50 channels on the ER surface. Finally, Ca^2+^ and *IP*_3_ binding and dissociation constants to *IP*_3_*R* were adjusted to fit our experimental data of calcium micro-domains in organotypic cultures of hippocampal astrocytes.
- A second variant of the 3d model, referred to as the “GCaMP”(=”GCaMP6s”) model, was obtained by adding GCaMP6s calcium indicators in the cytosol. GCaMP6s are ultrasensitive calcium indicators that fluoresce when bound to Ca^2+^. The fluorescence signal from experimental data indeed corresponds to the concentration of calcium-bound GCaMP6s, which can be quite different from free cytosolic Ca^2+^ trace. For this model, all the parameters were set to the same values as the “No-GCaMP” model, except those related to GCaMP that were taken from the available experimental literature and shown in Table 2. The parameters for GCaMP6f kinetics were taken from Chen et al. [72].
- A third variant of the 3d model, referred to as the “GC+Buf” model, was obtained by adding endogenous buffers to the “GCaMP” model. The kinetic scheme is presented in S2 Fig. Parameter values for calcium, IP_3_, IP_3_R and GCaMP dynamics were the same as the “GCaMP” model. Parameter values for endogenous buffers dynamics were taken from the literature [127] and are presented in S1 Table.

**Table 2:** Parameter values and initial conditions of the 3d model. The parameter values for the 3d model listed here correspond to the “GCaMP” model. The parameter values for the “No-GCaMP” 3d model are the same except that GCaMP6s concentration equals 0 nM. In the “GCaMP6f” model variant, *Gcamp*_*f*_ = 1.05 × 10^7^ *M*^−1^.*s*^−1^ and *Gcamp*_*b*_ = 3.93*s*^−1^. Parameter values in the 3d model have been adjusted to optimize the match with experimental data as described in the Methods section. Note that the values for calcium and *IP*_3_ binding or unbinding to *IP*_3_*R*, i.e. the *a*_*i*_’s and *b*_*j*_’s parameters below, are smaller in our model than in the literature, probably because our model is not cooperative. For GCaMP6s and GCaMP6f, we used the diffusion coefficient of calmodulin. The initial number of Ca^2+^ ions was adjusted so that the measured basal GCaMP6s-Ca concentration was around 300nM [86, 124].

Parameter	Description	Value in 3d GCaMP model	Reference
V	Cell volume	2.81 × 10^−17^ L	[128]
*IP*_3_ dynamics			
*IP*_0_	Initial *IP*_3_ number/conc	3 molec. i.e 177 nM	[85]
*D*_IP3_	*IP*_3_ diffusion	280 *μm*^2^.*s*^−1^	[107]
*N*_plc_	PLC*δ* number/conc.	1696 molec. i.e 100 *μ*M	[129]
*δ*	PLC*δ* max rate	1 s^−1^	-
*β*	*IP*_3_ decay	1.2 × 10^−4^ s^−1^	-
Ca^2+^ dynamics			
*Ca*_0_	Initial Ca^2+^ number/conc.	5 molec. i.e 295 nM	[86]
*D*_Ca_	Ca^2+^ diffusion	13 *μ*m^2^.s^−1^	[107]
*μ*	Ca^2+^ flux through open *IP*_3_*R*	6 × 10^3^ s^−1^	-
*γ*	cytosolic Ca^2+^ influx	1.5 × 10^−7^ s^−1^	-
*α*	Ca^2+^ decay rate	30 s^−1^	-
GCaMP6s			
*C*_GCa_	GCaMP6s conc.	169 molec. i.e 10 *μ*M	[130, 131]
*D*_GCaMP_	GCaMP6s diffusion	50 *μ*m^2^.s^−1^	[132]
*Gcamp*_*f*_	GCaMP6s Ca binding rate	7.78 × 10^6^ M^−1^.s^−1^	[72]
*Gcamp*_*b*_	GCaMP6s-Ca dissociation rate	1.12 s^−1^	[72]
*IP*_3_*R*			
$N_{{\text{IP}}_{\text{3}}\text{R}}$	*IP*_3_*R* number	50 molec.	[89]
$d_{{\text{IP}}_{\text{3}}\text{R}}$	*IP*_3_*R* interact. distance	1 mesh triangle	-
*IP*_3_*R* binding			
*a*_1_	First Ca	1.2 × 10^6^ M^−1^.s^−1^	-
*a*_2_	*IP*_3_	4.1 × 10^7^ M^−1^.s^−1^	-
*a*_3_	Second Ca	1.6 × 10^4^ M^−1^.s^−1^	-
*IP*_3_*R* dissociation			
*b*_1_	First Ca	50 s^−1^	-
*b*_2_	*IP*_3_	400 s^−1^	-
*b*_3_	Second Ca	100 *s*^−1^	-

#### Simulations code

The code of our ODE, Gillespie, Particle-based and STEPS models is available on ModelDB [133] at http://modeldb.yale.edu/247694.

#### Peak detection and analysis

Automated peak detection from model simulations was based on the statistics of baseline calcium trace. A histogram of Ca^2+^ trace was built with a bin size of 0.25 ions and the mode of this histogram was used to define baseline calcium. A peak initiation corresponded to the time step where calcium trace overcame a peak threshold defined as baseline + *nσ*_*Ca*_ where *σ*_*Ca*_ is the standard deviation of the above histogram. The value of *n* varied between 2 and 4 and was set by hand for each simulation, depending on its signal/noise ratio. The peak was considered terminated when the calcium trace decreased again below peak threshold. This implies that in case of a second calcium peak starting before the first one terminated, both events were considered as being part of the same peak. Peak duration was defined as the full width at half maximum (FWHM). Peak amplitude was defined as the maximum number of calcium ions reached during the peak duration. In the 3D model, the peak amplitude A was rescaled to facilitate comparison with experimental data, using $\Delta F/F=\left(A-C_{a_{\text{baseline}}}\right)/C_{a_{\text{baseline}}}$, where $C_{a_{\text{baseline}}}$ is the basal calcium determined above. The number of *IP*_3_*R* open per peak was defined as the maximum number of *IP*_3_*R* open simultaneously during peak duration. Puffs were defined as calcium events resulting from the cooperation of more than one *IP*_3_*R*. In our spatially-explicit simulations, a calcium signal was considered to be a puff if more than one *IP*_3_*R* were open during the peak and if the average distance traveled by calcium within the duration of this peak was larger than the distance between the simultaneously open *IP*_3_*R* molecules.

### Experimental methods

All experiments were performed as described in [69]. We give below the main outlines of the methods. All experimental procedures were in accordance with the European Union and CNRS UMR5297 institutional guidelines for the care and use of laboratory animals (Council directive 2010/63/EU).

#### Organotypic hippocampal slice cultures

Organotypic hippocampal slices (Gähwiler type) were dissected from 5–7-d-old wild-type mice and cultured 5–8 week in a roller drum at 35°C, as previously described [134].

#### Viral infection

AAV9-GFAP-GCaMP6s [70] was injected by brief pressure pulses (40ms; 15 psi) into the stratum radiatum of 2-3-week old slices from Thy1-YFP-H (JAX:003782) mice 4-6 weeks prior to the experiment.

#### Image acquisition

For Ca^2+^ imaging, we used a custom-built setup based on an inverted microscope body (Leica DMI6000), as previously described in [135]. We used a 1.3 NA glycerol immersion objective equipped with a correction collar to reduce spherical aberrations and thereby allow imaging deeper inside brain tissue [136]. The excitation light was provided by a pulsed diode laser (l = 485 nm, PicoQuant, Berlin, Germany). The fluorescence signal was confocally detected by an avalanche photodiode (APD; SPCM-AQRH-14-FC; PerkinElmer). The spatial resolution of the setup was around 175 nm (in x-y) and 450 nm (z). Confocal time-lapse imaging (12.5 x 25 *μ*m, pixel size 100 nm) was performed at 2Hz for 2.5 min in artificial cerebrospinal fluid containing 125 mM NaCl, 2.5 mM KCl, 1.3 mM MgCl_2_, 2 mM CaCl_2_, 26 mM NaHCO_3_, 1.25 mM NaH_2_PO_4_, 20 mM D-glucose, 1 mM Trolox; 300 mOsm; pH 7.4. Perfusion rate was 2 mL/min and the temperature 32°C. Calcium traces are available at https://figshare.com/articles/Astrocytic_calcium_traces_from_organotypic_hippocampal_slices/8951006.

#### Image analysis

Spontaneous calcium events were detected and analyzed automatically by ImageJ plugin LC_Pro [137] and then manually verified using Igor Pro (Wavemetrics) [33].

### Statistical analysis

For stochastic models, we generated 20 simulations (with different random numbers) and quantified these simulations as mean ± standard deviation over those 20 simulations. One-way ANOVA was performed to investigate the effect of a given parameter on calcium dynamics. Comparison between two simulation conditions were performed with unpaired Student T test if values followed a Gaussian distribution. Otherwise, a Mann-Whitney test was performed. The same method was applied to compare simulation to experimental results. Significance is assigned by * for *p* ≤ 0.05, ** for *p* ≤ 0.01, *** for *p* ≤ 0.001.

## Supporting information

S1 Fig Effect of adding buffers to the 2D particle-based model. (*A*) Biochemical reactions and regulatory interactions modeled in the 2D particle-based model in which endogenous buffers (’Buf’) were added. Reactions are the same as the ones described in Fig 1, except that Buf particles were added. The binding rate and dissociation constant associated with the binding of *Ca*^2+^ to Buf correspond respectively to *buf*_*f*_ and *buf*_*b*_. Different amounts of endogenous *Ca*^2+^ buffers were added to the model (500 or 2000), with the following diffusion coefficients: *D*_*buf*_ = 0.1 a.u and *D*_*Ca*_ = 0.8 a.u. Those simulations were compared to our reference model, which contains no Buf particles but in which *D*_*Ca*_ = 0.1 a.u, corresponding to an effective lower *D*_*Ca*_. No significant difference between simulations with a number of Buf of 0, 500 or 2000 is observed regarding basal *Ca*^2+^ concentration (B), peak amplitude (C) or peak frequency (D). Note that we refer here to free *Ca*^2+^ peaks and not to Buf-Ca peaks. Simulating *Ca*^2+^ diffusion in our 2D model with a decreased effective coefficient of diffusion is thus equivalent to simulating endogenous buffers of slower diffusion with faster diffusion of free *Ca*^2+^.
(PDF)

S2 Fig Reaction scheme of the 3D “GC+Buf” model. This figure presents the biochemical reactions and regulatory interactions modeled in the endogenous buffers model, “GC+Buf”, in 3D. Reactions are the same as the ones described in Fig 1, except that new particles have been added: slow (CBs) and fast calbindin (CBf) as well as parvalbumin (PV), that can bind *Ca*^2+^ ions and diffuse, whether bound or not. Parameter values associated with this model are presented in S1 Table.
(PDF)

S1 Table Parameter values and initial conditions associated to endogenous buffers in the 3D “GC+Buf” model. Parameter values for the “GC+Buf” 3d model described in supplemental S2 Fig are the same as in “GCaMP” model, presented in Table 2. Parameter values associated with endogenous kinetics and diffusion were taken from a study that modeled calcium dynamics in dendrites [127]. Note that total endogenous buffer concentration in our model is 2 orders of magnitude lower than in this study.
(PDF)

## References

[1] Sontheimer H. Voltage-dependent ion channels in glial cells. Glia. 1994;11(2):156–172. 10.1002/glia.440110210 7523291

[2] Orkand RK, Nicholls JG, Kuffler SW. Effect of nerve impulses on the membrane potential of glial cells in the central nervous system of amphibia. Journal of Neurophysiology. 1966;29(4):788–806. 10.1152/jn.1966.29.4.788 5966435

[3] Cornell-Bell A, Finkbeiner S, Cooper M, Smith S. Glutamate induces calcium waves in cultured astrocytes: long-range glial signaling. Science. 1990;247(4941):470–473. 1967852 10.1126/science.1967852

[4] Di Castro MA, Chuquet J, Liaudet N, Bhaukaurally K, Santello M, Bouvier D, et al Local Ca2+ detection and modulation of synaptic release by astrocytes. Nature Neuroscience. 2011;14(10):1276–1284. 10.1038/nn.2929 21909085

[5] Gómez-Gonzalo M, Navarrete M, Perea G, Covelo A, Martín-Fernández M, Shigemoto R, et al Endocannabinoids Induce Lateral Long-Term Potentiation of Transmitter Release by Stimulation of Gliotransmission. Cerebral Cortex (New York, NY: 1991). 2015;25(10):3699–3712. 10.1093/cercor/bhu231 25260706

[6] Panatier A, Theodosis DT, Mothet JP, Touquet B, Pollegioni L, Poulain DA, et al Glia-Derived d-Serine Controls NMDA Receptor Activity and Synaptic Memory. Cell. 2006;125(4):775–784. 10.1016/j.cell.2006.02.051 16713567

[7] Takata N, Mishima T, Hisatsune C, Nagai T, Ebisui E, Mikoshiba K, et al Astrocyte calcium signaling transforms cholinergic modulation to cortical plasticity in vivo. The Journal of Neuroscience: The Official Journal of the Society for Neuroscience. 2011;31(49):18155–18165. 10.1523/JNEUROSCI.5289-11.2011 22159127 PMC6634158

[8] Metea MR, Newman EA. Glial Cells Dilate and Constrict Blood Vessels: A Mechanism of Neurovascular Coupling. Journal of Neuroscience. 2006;26(11):2862–2870. 10.1523/JNEUROSCI.4048-05.2006 16540563 PMC2270788

[9] Mulligan S, MacVicar B.A Mulligan S.J. & MacVicar B.A. Calcium transients in astrocyte endfeet cause cerebrovascular constrictions. Nature 431, 195-199. Nature. 2004;431:195–9. 10.1038/nature02827 15356633

[10] Takano T, Tian GF, Peng W, Lou N, Libionka W, Han X, et al Astrocyte-mediated control of cerebral blood flow. Nature Neuroscience. 2006;9(2):260–267. 10.1038/nn1623 16388306

[11] Zonta M, Sebelin A, Gobbo S, Fellin T, Pozzan T, Carmignoto G. Glutamate-mediated cytosolic calcium oscillations regulate a pulsatile prostaglandin release from cultured rat astrocytes. The Journal of Physiology. 2003;553(Pt 2):407–414. 10.1113/jphysiol.2003.046706 14500777 PMC2343582

[12] Bazargani N, Attwell D. Astrocyte calcium signaling: the third wave. Nature Neuroscience. 2016;19(2):182–189. 10.1038/nn.4201 26814587

[13] Perea G, Navarrete M, Araque A. Tripartite synapses: astrocytes process and control synaptic information. Trends in Neurosciences. 2009;32(8):421–431. 10.1016/j.tins.2009.05.001 19615761

[14] Fiacco TA, McCarthy KD. Multiple Lines of Evidence Indicate That Gliotransmission Does Not Occur under Physiological Conditions. Journal of Neuroscience. 2018;38(1):3–13. 10.1523/JNEUROSCI.0016-17.2017 29298904 PMC5761435

[15] Savtchouk I, Volterra A. Gliotransmission: Beyond Black-and-White. Journal of Neuroscience. 2018;38(1):14–25. 10.1523/JNEUROSCI.0017-17.2017 29298905 PMC6705815

[16] Fellin T, Pascual O, Gobbo S, Pozzan T, Haydon PG, Carmignoto G. Neuronal Synchrony Mediated by Astrocytic Glutamate through Activation of Extrasynaptic NMDA Receptors. Neuron. 2004;43(5):729–743. 10.1016/j.neuron.2004.08.011 15339653

[17] Perea G, Araque A. Properties of synaptically evoked astrocyte calcium signal reveal synaptic information processing by astrocytes. The Journal of Neuroscience: The Official Journal of the Society for Neuroscience. 2005;25(9):2192–2203. 10.1523/JNEUROSCI.3965-04.2005 15745945 PMC6726085

[18] Morquette P, Verdier D, Kadala A, Féthière J, Philippe AG, Robitaille R, et al An astrocyte-dependent mechanism for neuronal rhythmogenesis. Nature Neuroscience. 2015;18(6):844–854. 10.1038/nn.4013 25938883

[19] Wang X, Lou N, Xu Q, Tian GF, Peng WG, Han X, et al Astrocytic Ca2+ signaling evoked by sensory stimulation in vivo. Nature Neuroscience. 2006;9(6):816–823. 10.1038/nn1703 16699507

[20] Asada A, Ujita S, Nakayama R, Oba S, Ishii S, Matsuki N, et al Subtle modulation of ongoing calcium dynamics in astrocytic microdomains by sensory inputs. Physiological Reports. 2015;3(10). 10.14814/phy2.12454 26438730 PMC4632942

[21] Haustein MD, Kracun S, Lu XH, Shih T, Jackson-Weaver O, Tong X, et al Conditions and constraints for astrocyte calcium signaling in the hippocampal mossy fiber pathway. Neuron. 2014;82(2):413–429. 10.1016/j.neuron.2014.02.041 24742463 PMC4086217

[22] Morita M, Higuchi C, Moto T, Kozuka N, Susuki J, Itofusa R, et al Dual Regulation of Calcium Oscillation in Astrocytes by Growth Factors and Pro-Inflammatory Cytokines via the Mitogen-Activated Protein Kinase Cascade. Journal of Neuroscience. 2003;23(34):10944–10952. 10.1523/JNEUROSCI.23-34-10944.2003 14645490 PMC6740971

[23] Nett WJ, Oloff SH, McCarthy KD. Hippocampal Astrocytes In Situ Exhibit Calcium Oscillations That Occur Independent of Neuronal Activity. Journal of Neurophysiology. 2002;87(1):528–537. 10.1152/jn.00268.2001 11784768

[24] Parri HR, Crunelli V. The role of Ca2+ in the generation of spontaneous astrocytic Ca2+ oscillations. Neuroscience. 2003;120(4):979–992. 10.1016/s0306-4522(03)00379-8 12927204

[25] Zur Nieden R, Deitmer JW. The role of metabotropic glutamate receptors for the generation of calcium oscillations in rat hippocampal astrocytes in situ. Cerebral Cortex (New York, NY: 1991). 2006;16(5):676–687. 10.1093/cercor/bhj013 16079243

[26] Panatier A, Vallée J, Haber M, Murai KK, Lacaille JC, Robitaille R. Astrocytes are endogenous regulators of basal transmission at central synapses. Cell. 2011;146(5):785–798. 10.1016/j.cell.2011.07.022 21855979

[27] Grosche J, Matyash V, Möller T, Verkhratsky A, Reichenbach A, Kettenmann H. Microdomains for neuron?glia interaction: parallel fiber signaling to Bergmann glial cells. Nature Neuroscience. 1999;2(2):139–143. 10.1038/5692 10195197

[28] Srinivasan R, Huang BS, Venugopal S, Johnston AD, Chai H, Zeng H, et al Ca(2+) signaling in astrocytes from Ip3r2(-/-) mice in brain slices and during startle responses in vivo. Nature Neuroscience. 2015;18(5):708–717. 10.1038/nn.4001 25894291 PMC4429056

[29] Beierlein M, Regehr WG. Brief Bursts of Parallel Fiber Activity Trigger Calcium Signals in Bergmann Glia. Journal of Neuroscience. 2006;26(26):6958–6967. 10.1523/JNEUROSCI.0613-06.2006 16807325 PMC6673913

[30] Matyash V, Filippov V, Mohrhagen K, Kettenmann H. Nitric Oxide Signals Parallel Fiber Activity to Bergmann Glial Cells in the Mouse Cerebellar Slice. Molecular and Cellular Neuroscience. 2001;18(6):664–670. 10.1006/mcne.2001.1047 11749041

[31] Newman EA. Propagation of Intercellular Calcium Waves in Retinal Astrocytes and Müller Cells. Journal of Neuroscience. 2001;21(7):2215–2223. 10.1523/JNEUROSCI.21-07-02215.2001 11264297 PMC2409971

[32] Cahoy JD, Emery B, Kaushal A, Foo LC, Zamanian JL, Christopherson KS, et al A Transcriptome Database for Astrocytes, Neurons, and Oligodendrocytes: A New Resource for Understanding Brain Development and Function. Journal of Neuroscience. 2008;28(1):264–278. 10.1523/JNEUROSCI.4178-07.2008 18171944 PMC6671143

[33] Sherwood MW, Arizono M, Hisatsune C, Bannai H, Ebisui E, Sherwood JL, et al Astrocytic IP3Rs: Contribution to Ca2+ signalling and hippocampal LTP. Glia. 2017;65(3):502–513. 10.1002/glia.23107 28063222

[34] Fiacco TA, Agulhon C, McCarthy KD. Sorting Out Astrocyte Physiology from Pharmacology. Annual Review of Pharmacology and Toxicology. 2009;49(1):151–174. 10.1146/annurev.pharmtox.011008.145602 18834310

[35] Porter JT, McCarthy KD. Astrocytic neurotransmitter receptors in situ and in vivo. Progress in Neurobiology. 1997;51(4):439–455. 10.1016/S0301-0082(96)00068-8 9106901

[36] Bosanac I, Alattia JR, Mal TK, Chan J, Talarico S, Tong FK, et al Structure of the inositol 1,4,5-trisphosphate receptor binding core in complex with its ligand. Nature. 2002;420(6916):696–700. 10.1038/nature01268 12442173

[37] Panatier A, Arizono M, Nägerl UV. Dissecting tripartite synapses with STED microscopy. Phil Trans R Soc B. 2014;369(1654):20130597 10.1098/rstb.2013.0597 25225091 PMC4173283

[38] Bindocci E, Savtchouk I, Liaudet N, Becker D, Carriero G, Volterra A. Three-dimensional Ca^2+^ imaging advances understanding of astrocyte biology. Science. 2017;356(6339):eaai8185 10.1126/science.aai8185 28522470

[39] Ventura R, Harris KM. Three-Dimensional Relationships between Hippocampal Synapses and Astrocytes. The Journal of Neuroscience. 1999;19(16):6897–6906. 10.1523/JNEUROSCI.19-16-06897.1999 10436047 PMC6782870

[40] Rusakov DA. Disentangling calcium-driven astrocyte physiology. Nature Reviews Neuroscience. 2015;16(4):226–233. 10.1038/nrn3878 25757560

[41] Thillaiappan NB, Chavda A, Tovey S, Prole D, Taylor C. Ca2+ signals initiate at immobile IP3 receptors adjacent to ER-plasma membrane junctions. Nature Communications. 2017;8 10.1038/s41467-017-01644-8 29138405 PMC5686115

[42] Smith IF, Wiltgen SM, Parker I. Localization of puff sites adjacent to the plasma membrane: Functional and spatial characterization of Ca2+ signaling in SH-SY5Y cells utilizing membrane-permeant caged IP3. Cell Calcium. 2009;45(1):65–76. 10.1016/j.ceca.2008.06.001 18639334 PMC2666303

[43] Manninen T, Havela R, Linne ML. Reproducibility and Comparability of Computational Models for Astrocyte Calcium Excitability. Frontiers in Neuroinformatics. 2017;11 10.3389/fninf.2017.00011 28270761 PMC5318440

[44] Oschmann F, Berry H, Obermayer K, Lenk K. From in silico astrocyte cell models to neuron-astrocyte network models: A review. Brain Research Bulletin. 2017 10.1016/j.brainresbull.2017.01.027 28189516

[45] Manninen T, Havela R, Linne ML. Computational Models for Calcium-Mediated Astrocyte Functions. Frontiers in Computational Neuroscience. 2018;12 10.3389/fncom.2018.00014 29670517 PMC5893839

[46] De Young GW, Keizer J. A single-pool inositol 1,4,5-trisphosphate-receptor-based model for agonist-stimulated oscillations in Ca2+ concentration. Proceedings of the National Academy of Sciences. 1992;89(20):9895–9899. 10.1073/pnas.89.20.9895 PMC50240 1329108

[47] Goldbetter A, Dupont G, Berridge MJ. Minimal Model for Signal-Induced Ca2+ Oscillations and for Their Frequency Encoding Through Protein Phosphorylation. Proceedings of the National Academy of Science. 1990;87:1461–1465. 10.1073/pnas.87.4.1461 PMC53495 2304911

[48] Li YX, Rinzel J. Equations for InsP3 Receptor-mediated [Ca2+]i Oscillations Derived from a Detailed Kinetic Model: A Hodgkin-Huxley Like Formalism. Journal of Theoretical Biology. 1994;166(4):461–473. 10.1006/jtbi.1994.1041 8176949

[49] Pittà MD, Goldberg M, Volman V, Berry H, Ben-Jacob E. Glutamate regulation of calcium and IP<Subscript>3</Subscript> oscillating and pulsating dynamics in astrocytes. Journal of Biological Physics. 2009;35(4):383–411. 10.1007/s10867-009-9155-y 19669422 PMC2750743

[50] Goto I, Kinoshita S, Natsume K. The model of glutamate-induced intracellular Ca2+ oscillation and intercellular Ca2+ wave in brain astrocytes. Neurocomputing—IJON. 2004;58:461–467.

[51] Falcke M, Tsimring L, Levine H. Stochastic spreading of intracellular Ca(2+) release. Physical Review E, Statistical Physics, Plasmas, Fluids, and Related Interdisciplinary Topics. 2000;62(2 Pt B):2636–2643. 11088743 10.1103/physreve.62.2636

[52] Rüdiger S, Shuai JW, Huisinga W, Nagaiah C, Warnecke G, Parker I, et al Hybrid stochastic and deterministic simulations of calcium blips. Biophysical Journal. 2007;93(6):1847–1857. 10.1529/biophysj.106.099879 17496042 PMC1959544

[53] Rüdiger S, Nagaiah C, Warnecke G, Shuai JW. Calcium Domains around Single and Clustered IP3 Receptors and Their Modulation by Buffers. Biophysical Journal. 2010;99(1):3–12. 10.1016/j.bpj.2010.02.059 20655827 PMC2895387

[54] Skupin A, Kettenmann H, Falcke M. Calcium Signals Driven by Single Channel Noise. PLoS computational biology. 2010;6 10.1371/journal.pcbi.1000870 20700497 PMC2917103

[55] Dobramysl U, Rüdiger S, Erban R. Particle-Based Multiscale Modeling of Calcium Puff Dynamics. Multiscale Modeling & Simulation. 2016; p. 997–1016. 10.1137/15M1015030

[56] Bezprozvanny I, Watras J, Ehrlich BE. Bell-shaped calcium-response curves of Ins(1,4,5)P3- and calcium-gated channels from endoplasmic reticulum of cerebellum. Nature. 1991;351(6329):751–754. 10.1038/351751a0 1648178

[57] Swillens S, Dupont G, Combettes L, Champeil P. From calcium blips to calcium puffs: theoretical analysis of the requirements for interchannel communication. Proceedings of the National Academy of Sciences of the United States of America. 1999;96(24):13750–13755. 10.1073/pnas.96.24.13750 10570144 PMC24136

[58] Smith IF, Wiltgen SM, Shuai J, Parker I. Ca2+ Puffs Originate from Preestablished Stable Clusters of Inositol Trisphosphate Receptors. Sci Signal. 2009;2(98):ra77–ra77. 10.1126/scisignal.2000466 19934435 PMC2897231

[59] Dickinson G, Swaminathan D, Parker I. The Probability of Triggering Calcium Puffs Is Linearly Related to the Number of Inositol Trisphosphate Receptors in a Cluster. Biophysical Journal. 2012;102(8):1826–1836. 10.1016/j.bpj.2012.03.029 22768938 PMC3328715

[60] Flegg MB, Rüdiger S, Erban R. Diffusive spatio-temporal noise in a first-passage time model for intracellular calcium release. The Journal of Chemical Physics. 2013;138(15):154103 10.1063/1.4796417 23614408

[61] Pando B, Dawson SP, Mak DOD, Pearson JE. Messages diffuse faster than messengers. Proceedings of the National Academy of Sciences. 2006;103(14):5338–5342. 10.1073/pnas.0509576103 PMC1414799 16569700

[62] Fraiman D, Dawson SP. Buffer regulation of calcium puff sequences. Physical Biology. 2014;11(1):016007 10.1088/1478-3975/11/1/016007 24476691

[63] Falcke M. Buffers and oscillations in intracellular Ca2+ dynamics. Biophysical Journal. 2003;84(1):28–41. 10.1016/S0006-3495(03)74830-9 12524263 PMC1302591

[64] Shuai J, Pearson JE, Parker I. Modeling Ca2+ Feedback on a Single Inositol 1,4,5-Trisphosphate Receptor and Its Modulation by Ca2+ Buffers. Biophysical Journal. 2008;95(8):3738–3752. 10.1529/biophysj.108.137182 18641077 PMC2553123

[65] Lencesova L, O’Neill A, Resneck WG, Bloch RJ, Blaustein MP. Plasma membrane-cytoskeleton-endoplasmic reticulum complexes in neurons and astrocytes. The Journal of Biological Chemistry. 2004;279(4):2885–2893. 10.1074/jbc.M310365200 14593108

[66] Weerth SH, Holtzclaw LA, Russell JT. Signaling proteins in raft-like microdomains are essential for Ca2+ wave propagation in glial cells. Cell Calcium. 2007;41(2):155–167. 10.1016/j.ceca.2006.06.006 16905188

[67] Buscemi L, Ginet V, Lopatar J, Montana V, Pucci L, Spagnuolo P, et al Homer1 Scaffold Proteins Govern Ca2+ Dynamics in Normal and Reactive Astrocytes. Cerebral Cortex (New York, NY: 1991). 2016. 10.1093/cercor/bhw078 PMC5963825 27075036

[68] Xiao B, Tu JC, Worley PF. Homer: a link between neural activity and glutamate receptor function. Current Opinion in Neurobiology. 2000;10(3):370–374. 10.1016/S0959-4388(00)00087-8 10851183

[69] Arizono M, Panatier A, Inavalli VVGK, Pfeiffer T, Angibaud J, Stobart J, et al Structural Basis of Astrocytic Ca ^2^ Signals at Tripartite Synapses. Rochester, NY: Social Science Research Network; 2018 ID 3287791. Available from: https://papers.ssrn.com/abstract=3287791.

[70] Stobart JL, Ferrari KD, Barrett MJP, Stobart MJ, Looser ZJ, Saab AS, et al Long-term In Vivo Calcium Imaging of Astrocytes Reveals Distinct Cellular Compartment Responses to Sensory Stimulation. Cerebral Cortex (New York, NY: 1991). 2018;28(1):184–198. 10.1093/cercor/bhw366 28968832

[71] Skupin A, Kettenmann H, Winkler U, Wartenberg M, Sauer H, Tovey SC, et al How Does Intracellular Ca2+ Oscillate: By Chance or by the Clock? Biophysical Journal. 2008;94(6):2404–2411. 10.1529/biophysj.107.119495 18065468 PMC2257893

[72] Chen TW, Wardill TJ, Sun Y, Pulver SR, Renninger SL, Baohan A, et al Ultrasensitive fluorescent proteins for imaging neuronal activity. Nature. 2013;499(7458):295–300. 10.1038/nature12354 23868258 PMC3777791

[73] Patrushev I, Gavrilov N, Turlapov V, Semyanov A. Subcellular location of astrocytic calcium stores favors extrasynaptic neuron-astrocyte communication. Cell Calcium. 2013;54(5):343–349. 10.1016/j.ceca.2013.08.003 24035346

[74] Montes de Oca Balderas P, Montes de Oca Balderas H. Synaptic neuron-astrocyte communication is supported by an order of magnitude analysis of inositol tris-phosphate diffusion at the nanoscale in a model of peri-synaptic astrocyte projection. BMC Biophysics. 2018;11:3 10.1186/s13628-018-0043-3 29456837 PMC5809920

[75] Boulay AC, Saubaméa B, Adam N, Chasseigneaux S, Mazaré N, Gilbert A, et al Translation in astrocyte distal processes sets molecular heterogeneity at the gliovascular interface. Cell Discovery. 2017;3:17005 10.1038/celldisc.2017.5 28377822 PMC5368712

[76] Jones VC, McKeown L, Verkhratsky A, Jones OT. LV-pIN-KDEL: a novel lentiviral vector demonstrates the morphology, dynamics and continuity of the endoplasmic reticulum in live neurones. BMC Neuroscience. 2008;9:10 10.1186/1471-2202-9-10 18215281 PMC2248189

[77] Nixon-Abell J, Obara CJ, Weigel AV, Li D, Legant WR, Xu CS, et al Increased spatiotemporal resolution reveals highly dynamic dense tubular matrices in the peripheral ER. Science. 2016;354(6311):aaf3928 10.1126/science.aaf3928 27789813 PMC6528812

[78] Brunstein M, Wicker K, Hérault K, Heintzmann R, Oheim M. Full-field dual-color 100-nm super-resolution imaging reveals organization and dynamics of mitochondrial and ER networks. Optics Express. 2013;21(22):26162–26173. 10.1364/OE.21.026162 24216840

[79] Kopek BG, Paez-Segala MG, Shtengel G, Sochacki KA, Sun MG, Wang Y, et al Diverse protocols for correlative super-resolution fluorescence imaging and electron microscopy of chemically fixed samples. Nature protocols. 2017;12(5):916–946. 10.1038/nprot.2017.017 28384138 PMC5514615

[80] Okubo Y, Kanemaru K, Suzuki J, Kobayashi K, Hirose K, Iino M. Inositol 1,4,5-trisphosphate receptor type 2-independent Ca2+ release from the endoplasmic reticulum in astrocytes. Glia. 2019;67(1):113–124. 10.1002/glia.23531 30306640

[81] Bannai H, Hirose M, Niwa F, Mikoshiba K. Dissection of Local Ca2+ Signals in Cultured Cells by Membrane targeted Ca2+ Indicators. JoVE (Journal of Visualized Experiments). 2019;(145):e59246. 10.3791/59246 30958464

[82] Alzayady KJ, Sebé-Pedrós A, Chandrasekhar R, Wang L, Ruiz-Trillo I, Yule DI. Tracing the Evolutionary History of Inositol, 1, 4, 5-Trisphosphate Receptor: Insights from Analyses of Capsaspora owczarzaki Ca2+ Release Channel Orthologs. Molecular Biology and Evolution. 2015;32(9):2236–2253. 10.1093/molbev/msv098 25911230 PMC4540961

[83] Serysheva II, Baker MR, Fan G. Structural Insights into IP<Subscript>3</Subscript>R Function In: Membrane Dynamics and Calcium Signaling. Advances in Experimental Medicine and Biology. Springer, Cham; 2017 p. 121–147. Available from: https://link.springer.com/chapter/10.1007/978-3-319-55858-5_6. 10.1007/978-3-319-55858-5_6 29594860

[84] Bicknell BA, Goodhill GJ. Emergence of ion channel modal gating from independent subunit kinetics. Proceedings of the National Academy of Sciences. 2016;113(36):E5288–E5297. 10.1073/pnas.1604090113 PMC5018786 27551100

[85] Oura T, Murata K, Morita T, Nezu A, Arisawa M, Shuto S, et al Highly Sensitive Measurement of Inositol 1,4,5-Trisphosphate by Using a New Fluorescent Ligand and Ligand Binding Domain Combination. Chembiochem: A European Journal of Chemical Biology. 2016;17(16):1509–1512. 10.1002/cbic.201600096 27251449

[86] Zheng K, Bard L, Reynolds JP, King C, Jensen T, Gourine A, et al Time-Resolved Imaging Reveals Heterogeneous Landscapes of Nanomolar Ca2+ in Neurons and Astroglia. Neuron. 2015;88(2):277–288. 10.1016/j.neuron.2015.09.043 26494277 PMC4622934

[87] Gin E, Falcke M, Wagner LE, Yule DI, Sneyd J. A Kinetic Model of the Inositol Trisphosphate Receptor Based on Single-Channel Data. Biophysical Journal. 2009;96(10):4053–4062. 10.1016/j.bpj.2008.12.3964 19450477 PMC2712151

[88] Siekmann I, Wagner L, Yule D, Crampin E, Sneyd J. A Kinetic Model for Type I and II IP3R Accounting for Mode Changes. Biophysical Journal. 2012;103(4):658–668. 10.1016/j.bpj.2012.07.016 22947927 PMC3443778

[89] Wiltgen SM, Smith IF, Parker I. Superresolution localization of single functional IP3R channels utilizing Ca2+ flux as a readout. Biophysical Journal. 2010;99(2):437–446. 20643061 10.1016/j.bpj.2010.04.037 PMC2905071

[90] Tay LH, Dick IE, Yang W, Mank M, Griesbeck O, Yue DT. Nanodomain Ca^2+^ of Ca^2+^ channels detected by a tethered genetically encoded Ca^2+^ sensor. Nature Communications. 2012;3:778 10.1038/ncomms1777 22491326 PMC3615648

[91] Tadross MR, Tsien RW, Yue DT. Ca2+ channel nanodomains boost local Ca2+ amplitude. Proceedings of the National Academy of Sciences of the United States of America. 2013;110(39):15794–15799. 10.1073/pnas.1313898110 24019485 PMC3785779

[92] Sun MY, Devaraju P, Xie AX, Holman I, Samones E, Murphy TR, et al Astrocyte calcium microdomains are inhibited by Bafilomycin A1 and cannot be replicated by low-level Schaffer collateral stimulation in situ. Cell Calcium. 2014;55(1):1–16. 10.1016/j.ceca.2013.10.004 24262208

[93] Agarwal A, Wu PH, Hughes EG, Fukaya M, Tischfield MA, Langseth AJ, et al Transient Opening of the Mitochondrial Permeability Transition Pore Induces Microdomain Calcium Transients in Astrocyte Processes. Neuron. 2017;93(3):587–605.e7. 10.1016/j.neuron.2016.12.034 28132831 PMC5308886

[94] Mauvezin C, Neufeld TP. Bafilomycin A1 disrupts autophagic flux by inhibiting both V-ATPase-dependent acidification and Ca-P60A/SERCA-dependent autophagosome-lysosome fusion. Autophagy. 2015;11(8):1437–1438. 10.1080/15548627.2015.1066957 26156798 PMC4590655

[95] Roest G, La Rovere RM, Bultynck G, Parys JB. IP3 Receptor Properties and Function at Membrane Contact Sites. Advances in Experimental Medicine and Biology. 2017;981:149–178. 10.1007/978-3-319-55858-5_7 29594861

[96] Wilson BS, Pfeiffer JR, Smith AJ, Oliver JM, Oberdorf JA, Wojcikiewicz RJH. Calcium-dependent Clustering of Inositol 1,4,5-Trisphosphate Receptors. Molecular Biology of the Cell. 1998;9(6):1465–1478. 10.1091/mbc.9.6.1465 9614187 PMC25370

[97] Rahman T. Dynamic clustering of IP _3_ receptors by IP_3_. Biochemical Society Transactions. 2012;40(2):325–330. 10.1042/BST20110772 22435806

[98] Smith I, Swaminathan D, Dickinson G, Parker I. Single-Molecule Tracking of Inositol Trisphosphate Receptors Reveals Different Motilities and Distributions. Biophysical Journal. 2014;107(4):834–845. 10.1016/j.bpj.2014.05.051 25140418 PMC4142249

[99] Shuai JW, Jung P. Optimal ion channel clustering for intracellular calcium signaling. Proceedings of the National Academy of Sciences. 2003;100(2):506–510. 10.1073/pnas.0236032100 PMC141025 12518049

[100] Skupin A, Falcke M. The role of IP3R clustering in Ca2+ signaling. Genome Informatics International Conference on Genome Informatics. 2008;20:15–24. 19425119

[101] Means S, Smith AJ, Shepherd J, Shadid J, Fowler J, Wojcikiewicz RJH, et al Reaction Diffusion Modeling of Calcium Dynamics with Realistic ER Geometry. Biophysical Journal. 2006;91(2):537–557. 10.1529/biophysj.105.075036 16617072 PMC1483115

[102] Tu H, Wang Z, Bezprozvanny I. Modulation of Mammalian Inositol 1,4,5-Trisphosphate Receptor Isoforms by Calcium: A Role of Calcium Sensor Region. Biophysical Journal. 2005;88(2):1056–1069. 10.1529/biophysj.104.049601 15531634 PMC1305112

[103] Tu H, Wang Z, Nosyreva E, De Smedt H, Bezprozvanny I. Functional Characterization of Mammalian Inositol 1,4,5-Trisphosphate Receptor Isoforms. Biophysical Journal. 2005;88(2):1046–1055. 10.1529/biophysj.104.049593 15533917 PMC1305111

[104] Taylor CW. Regulation of IP3 receptors by cyclic AMP. Cell Calcium. 2017;63:48–52. 10.1016/j.ceca.2016.10.005 27836216 PMC5471599

[105] Thrower EC, Hagar RE, Ehrlich BE. Regulation of Ins(1,4,5)P3 receptor isoforms by endogenous modulators. Trends in Pharmacological Sciences. 2001;22(11):580–586. 10.1016/S0165-6147(00)01809-5 11698102

[106] Wagner LE, Yule DI. Differential regulation of the InsP? receptor type-1 and -2 single channel properties by InsP3, Ca2+ and ATP. The Journal of Physiology. 2012;590(14):3245–3259. 10.1113/jphysiol.2012.228320 22547632 PMC3459040

[107] Allbritton NL, Meyer T, Stryer L. Range of messenger action of calcium ion and inositol 1,4,5-trisphosphate. Science (New York, NY). 1992;258(5089):1812–1815. 10.1126/science.1465619 1465619

[108] Wang Z, Tymianski M, Jones OT, Nedergaard M. Impact of Cytoplasmic Calcium Buffering on the Spatial and Temporal Characteristics of Intercellular Calcium Signals in Astrocytes. Journal of Neuroscience. 1997;17(19):7359–7371. 10.1523/JNEUROSCI.17-19-07359.1997 9295382 PMC6573438

[109] Zeller S, Rüdiger S, Engel H, Sneyd J, Warnecke G, Parker I, et al Modeling of the modulation by buffers of Ca2+ release through clusters of IP3 receptors. Biophysical Journal. 2009;97(4):992–1002. 10.1016/j.bpj.2009.05.050 19686646 PMC2726323

[110] Wieder N, Fink R, von Wegner F. Exact Stochastic Simulation of a Calcium Microdomain Reveals the Impact of Ca2+ Fluctuations on IP3R Gating. Biophysical Journal. 2015;108(3):557–567. 10.1016/j.bpj.2014.11.3458 25650923 PMC4317541

[111] Schwaller B. Cytosolic Ca2+ buffers. Cold Spring Harbor Perspectives in Biology. 2010;2(11):a004051 10.1101/cshperspect.a004051 20943758 PMC2964180

[112] Chai H, Diaz-Castro B, Shigetomi E, Monte E, Octeau JC, Yu X, et al Neural Circuit-Specialized Astrocytes: Transcriptomic, Proteomic, Morphological, and Functional Evidence. Neuron. 2017;95(3):531–549.e9. 10.1016/j.neuron.2017.06.029 28712653 PMC5811312

[113] Haim LB, Rowitch DH. Functional diversity of astrocytes in neural circuit regulation. Nature Reviews Neuroscience. 2017;18(1):31–41. 10.1038/nrn.2016.159 27904142

[114] Breslin K, Wade JJ, Wong-Lin K, Harkin J, Flanagan B, Zalinge HV, et al Potassium and sodium microdomains in thin astroglial processes: A computational model study. PLOS Computational Biology. 2018;14(5):e1006151 10.1371/journal.pcbi.1006151 29775457 PMC5979043

[115] Khalid MU, Tervonen A, Korkka I, Hyttinen J, Lenk K. Geometry-based Computational Modeling of Calcium Signaling in an Astrocyte In: EMBEC & NBC 2017. IFMBE Proceedings. Springer, Singapore; 2017 p. 157–160. Available from: https://link.springer.com/chapter/10.1007/978-981-10-5122-7_40.

[116] Savtchenko LP, Bard L, Jensen TP, Reynolds JP, Kraev I, Medvedev N, et al Disentangling astroglial physiology with a realistic cell model in silico. Nature Communications. 2018;9(1):3554 10.1038/s41467-018-05896-w 30177844 PMC6120909

[117] Gillespie DT. Exact stochastic simulation of coupled chemical reactions. The Journal of Physical Chemistry. 1977;81(25):2340–2361. 10.1021/j100540a008

[118] Gillespie DT. Stochastic simulation of chemical kinetics. Annual Review of Physical Chemistry. 2007;58:35–55. 10.1146/annurev.physchem.58.032806.104637 17037977

[119] Isaacson SA, Isaacson D. The Reaction-Diffusion Master Equation, Diffusion Limited Reactions, and Singular Potentials. Physical Review E, Statistical, Nonlinear, and Soft Matter Physics. 2009;80(6 Pt 2):066106 10.1103/PhysRevE.80.066106 20365230 PMC3405976

[120] Smith S, Grima R. Spatial Stochastic Intracellular Kinetics: A Review of Modelling Approaches. Bulletin of Mathematical Biology. 2018. 10.1007/s11538-018-0443-1 PMC6677717 29785521

[121] Hepburn I, Chen W, Wils S, De Schutter E. STEPS: efficient simulation of stochastic reaction?diffusion models in realistic morphologies. BMC Systems Biology. 2012;6(1):36 10.1186/1752-0509-6-36 22574658 PMC3472240

[122] Wils S, De Schutter E. STEPS: Modeling and Simulating Complex Reaction-Diffusion Systems with Python. Frontiers in Neuroinformatics. 2009;3:15 10.3389/neuro.11.015.2009 19623245 PMC2706651

[123] Chen W, De Schutter E. Parallel STEPS: Large Scale Stochastic Spatial Reaction-Diffusion Simulation with High Performance Computers. Frontiers in Neuroinformatics. 2017;11 10.3389/fninf.2017.00013 28239346 PMC5301017

[124] Shigetomi E, Patel S, Khakh BS. Probing the Complexities of Astrocyte Calcium Signaling. Trends in Cell Biology. 2016;26(4):300–312. 10.1016/j.tcb.2016.01.003 26896246 PMC4946798

[125] Swaminathan D, Jung P. The Role of agonist-independent conformational transformation (AICT) in IP3 cluster behavior. Cell Calcium. 2011;49(3):145–152. 10.1016/j.ceca.2010.11.003 21334066

[126] Hituri K, Linne ML. Comparison of Models for IP3 Receptor Kinetics Using Stochastic Simulations. PLOS ONE. 2013;8(4):e59618 10.1371/journal.pone.0059618 23630568 PMC3629942

[127] Anwar H, Hepburn I, Nedelescu H, Chen W, Schutter ED. Stochastic Calcium Mechanisms Cause Dendritic Calcium Spike Variability. Journal of Neuroscience. 2013;33(40):15848–15867. 10.1523/JNEUROSCI.1722-13.2013 24089492 PMC6618479

[128] Calì C, Baghabra J, Boges DJ, Holst GR, Kreshuk A, Hamprecht FA, et al Three-dimensional immersive virtual reality for studying cellular compartments in 3D models from EM preparations of neural tissues. Journal of Comparative Neurology. 2016;524(1):23–38. 10.1002/cne.23852 26179415 PMC5042088

[129] Hernàndez-Sotomayor SMT, Santos-Briones CDL, Muñoz-Sànchez JA, Loyola-Vargas VM. Kinetic Analysis of Phospholipase C from Catharanthus roseus Transformed Roots Using Different Assays. Plant Physiology. 1999;120(4):1075–1082. 10.1104/pp.120.4.1075 10444091 PMC59341

[130] Akerboom J, Chen TW, Wardill TJ, Tian L, Marvin JS, Mutlu S, et al Optimization of a GCaMP calcium indicator for neural activity imaging. The Journal of Neuroscience: The Official Journal of the Society for Neuroscience. 2012;32(40):13819–13840. 10.1523/JNEUROSCI.2601-12.2012 23035093 PMC3482105

[131] Hires SA, Tian L, Looger LL. Reporting neural activity with genetically encoded calcium indicators. Brain Cell Biology. 2008;36(1-4):69 10.1007/s11068-008-9029-4 18941901 PMC2755531

[132] Michailova A, Del Principe F, Egger M, Niggli E. Spatiotemporal Features of Ca2+ Buffering and Diffusion in Atrial Cardiac Myocytes with Inhibited Sarcoplasmic Reticulum. Biophysical journal. 2003;83:3134–51. 10.1016/S0006-3495(02)75317-4 PMC1302392 12496084

[133] McDougal RA, Morse TM, Carnevale T, Marenco L, Wang R, Migliore M, et al Twenty years of ModelDB and beyond: building essential modeling tools for the future of neuroscience. Journal of Computational Neuroscience. 2017;42(1):1–10. 10.1007/s10827-016-0623-7 27629590 PMC5279891

[134] Gähwiler BH. Organotypic monolayer cultures of nervous tissue. Journal of Neuroscience Methods. 1981;4(4):329–342. 10.1016/0165-0270(81)90003-0 7033675

[135] Tønnesen J, Nadrigny F, Willig K, Wedlich-Söldner R, Nägerl UV. Two-Color STED Microscopy of Living Synapses Using A Single Laser-Beam Pair. Biophysical Journal. 2011;101(10):2545–2552. 10.1016/j.bpj.2011.10.011 22098754 PMC3218326

[136] Urban N, Willig K, Hell S, Nägerl UV. STED Nanoscopy of Actin Dynamics in Synapses Deep Inside Living Brain Slices. Biophysical Journal. 2011;101(5):1277–1284. 10.1016/j.bpj.2011.07.027 21889466 PMC3164186

[137] Francis M, Waldrup J, Qian X, Taylor MS. Automated Analysis of Dynamic Ca2+ Signals in Image Sequences. Journal of Visualized Experiments: JoVE. 2014;(88). 10.3791/51560 PMC4195352 24962784

