# Supplementary figures and images for: Simulation of calcium signaling in fine astrocytic processes: Effect of spatial properties on spontaneous activity
Source: PLoS Comput Biol. 2019 Aug 19;15(8):e1006795. doi: 10.1371/journal.pcbi.1006795 (PMC6726244; doi:10.1371/journal.pcbi.1006795)

A

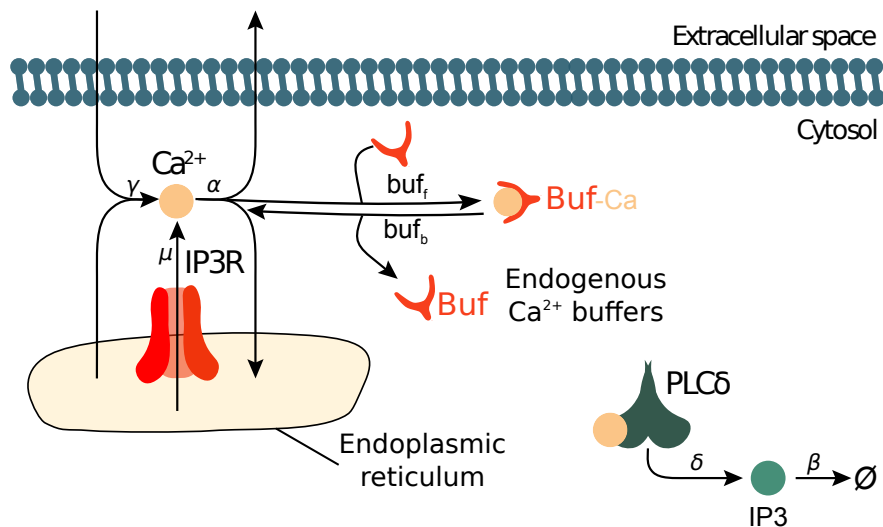

B

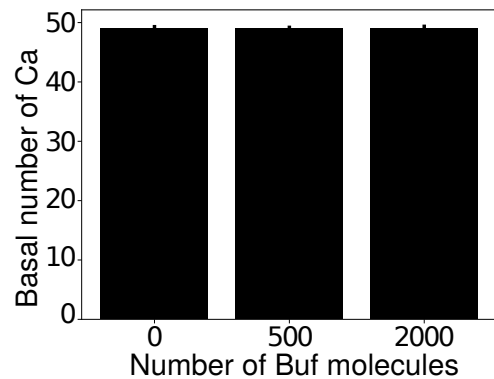

C

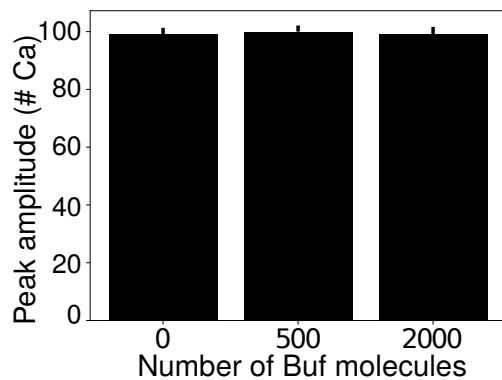

D

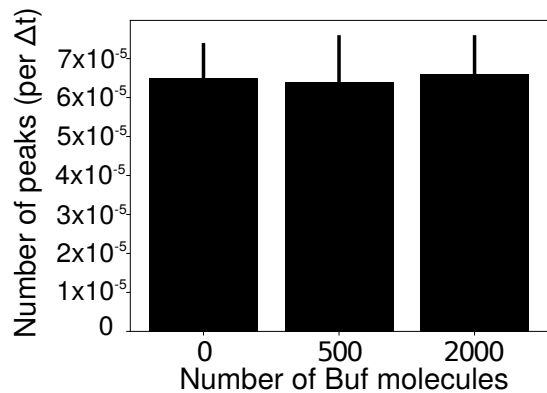

Supplement: S1 Fig — (A) Biochemical reactions and regulatory interactions modeled in the 2D particle-based model in which endogenous buffers (’Buf’) were added. Reactions are the same as the ones described in Fig 1, except that Buf particles were added. The binding rate and dissociation constant associated with the binding of Ca2+ to Buf correspond respectively to buff and bufb. Different amounts of endogenous Ca2+ buffers were added to the model (500 or 2000), with the following diffusion coefficients: Dbuf = 0.1 a.u and DCa = 0.8 a.u. Those simulations were compared to our reference model, which contains no Buf particles but in which DCa = 0.1 a.u, corresponding to an effective lower DCa. No significant difference between simulations with a number of Buf of 0, 500 or 2000 is observed regarding basal Ca2+ concentration (B), peak amplitude (C) or peak frequency (D). Note that we refer here to free Ca2+ peaks and not to Buf-Ca peaks. Simulating Ca2+ diffusion in our 2D model with a decreased effective coefficient of diffusion is thus equivalent to simulating endogenous buffers of slower diffusion with faster diffusion of free Ca2+. (PDF) [file pcbi.1006795.s001.pdf]

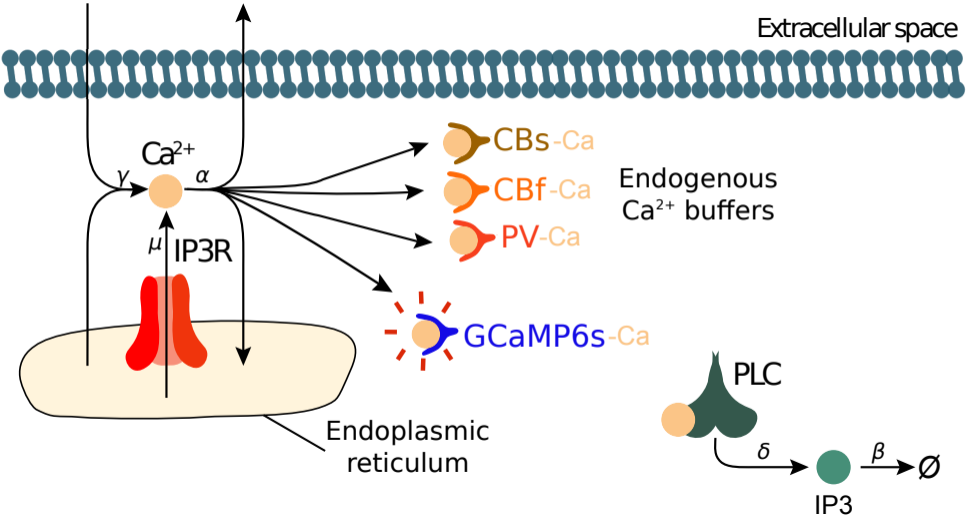

Supplement: S2 Fig — This figure presents the biochemical reactions and regulatory interactions modeled in the endogenous buffers model, “GC+Buf”, in 3D. Reactions are the same as the ones described in Fig 1, except that new particles have been added: slow (CBs) and fast calbindin (CBf) as well as parvalbumin (PV), that can bind Ca2+ ions and diffuse, whether bound or not. Parameter values associated with this model are presented in S1 Table. (PDF) [file pcbi.1006795.s002.pdf]
