# Supplementary material for: Simulation of calcium signaling in fine astrocytic processes: Effect of spatial properties on spontaneous activity
Source: PLoS Comput Biol. 2019 Aug 19;15(8):e1006795. doi: 10.1371/journal.pcbi.1006795 (PMC6726244; doi:10.1371/journal.pcbi.1006795)
Supplement: S1 Table — Parameter values for the “GC+Buf” 3d model described in supplemental S2 Fig are the same as in “GCaMP” model, presented in Table 2. Parameter values associated with endogenous kinetics and diffusion were taken from a study that modeled calcium dynamics in dendrites [127]. Note that total endogenous buffer concentration in our model is 2 orders of magnitude lower than in this study. (PDF) [file pcbi.1006795.s003.pdf]

Parameter	Description	Value in 3d	Reference
C_{PV}	Initial PV conc.	260 nM	[125]
C_{PV}	Initial Ca-PV conc.	400 nM	[125]
D_{PV}	PV diffusion	43 μm^2.s^{-1}	[125]
PV_f	PV Ca binding rate	10.7×10^7 M^{-1}.s^{-1}	[125]
PV_b	PV-Ca dissociation rate	0.95 s^{-1}	[125]
C_{CBs}	Initial CBs conc.	110 nM	[125]
C_{CBsCa}	Initial CBsCa conc.	200 nM	[125]
C_{CBsCa2}	Initial CBsCa2 conc.	200 nM	[125]
D_{CBs}	CBs diffusion	28 μm^2.s^{-1}	[125]
CBs_f	CBs Ca binding rate	5.5×10^6 M^{-1}.s^{-1}	[125]
CBs_b	CBs-Ca dissociation rate	2.6 s^{-1}	[125]
$CBsCa_f$	CBs-Ca Ca binding rate	5.5×10^6 M^{-1}.s^{-1}	[125]
$CBsCa_b$	CBs-Ca2 dissociation rate	2.6 s^{-1}	[125]
C_{CBf}	Initial CBf conc.	110 nM	[125]
C_{CBfCa}	Initial CBfCa conc.	200 nM	[125]
C_{CBfCa2}	Initial CBfCa2 conc.	200 nM	[125]
D_{CBf}	CBf diffusion	28 μm^2.s^{-1}	[125]
CBf_f	CBf Ca binding rate	4.35×10^7 M^{-1}.s^{-1}	[125]
CBf_b	CBf-Ca dissociation rate	35.8 s^{-1}	[125]
$CBfCa_f$	CBf-Ca Ca binding rate	4.35×10^7 M^{-1}.s^{-1}	[125]
$CBfCa_b$	CBf-Ca2 dissociation rate	35.8 s^{-1}	[125]
